# Systematic Evaluation of Toxicity of Aconite Based on Bibliometric Method

**DOI:** 10.1155/2021/5514281

**Published:** 2021-08-02

**Authors:** Diyao Wu, Tielong Xu, Zhendong Huang, Yaling Wang, Hongfu Chen, Qian Chen, Lihua Chen, Meiying Ao

**Affiliations:** ^1^College of Traditional Chinese Medicine, Jiangxi University of Traditional Chinese Medicine, Nanchang, Jiangxi 330004, China; ^2^College of Pharmacy, Jiangxi University of Traditional Chinese Medicine, Nanchang, Jiangxi 330004, China

## Abstract

**Aim:**

Based on the bibliometric method, the toxicity of aconite is analyzed and evaluated.

**Methods:**

Studies on the toxicity of aconite were retrieved from CNKI, CQVIP, Chinese Biomedical Literature Service System, and PubMed, ranging from January 1985 to November 2020. All those studies were formed into the Database of Literature of Toxicity of Aconite (DLTA). Studies on the toxicity of aconite were retrieved from CNKI, CQVIP, SinoMed, and PubMed, respectively. Collecting relevant information in DLTA, we analyzed the hotspots, factors and mechanism of aconite toxicity, and attenuation methods.

**Results:**

A total of 445 studies on the toxicity of aconite have been collected. “Compatibility attenuation” and “Processing attenuation” have been the hotspots of aconite toxicity in recent years. Many studies support that the main toxic reactions of aconite are heart damage, liver toxicity, nephrotoxicity, and neurotoxicity. The toxic effect of aconite is related to the effect on the central nervous system. Exciting the vagus nerve reduces the autonomy of the sinus node and damages myocardial cells. The decoction time, dosage, and administration of aconite are the main factors of the toxicity of aconite. There are few studies about the effect of the origin of aconite and the specifications of the medicinal materials on toxicity. Therefore, it is impossible to analyze its relevance. At present, the commonly used methods to reduce the toxicity of aconite mainly include three methods: drug compatibility, processing, and decoction. The most common compatibility with aconite medicines includes licorice, dried ginger, ginseng, and ephedra. Black sliced aconite, steamed slices, and fried slices are less toxic than other processed products. Aconite decoction for more than 60 minutes can basically reach the safe range, and more than 2 hours of decoction may cause the loss of active ingredients.

**Conclusions:**

The research on the mechanisms of aconite dosage-efficacy-toxicity, compatibility, processing, liver toxicity, and nephrotoxicity is still not comprehensive and in-depth. Researchers should perfect toxicity studies of aconite, remove the constraints that affect its clinical application, and promote the clinical use of aconite safely and reasonably.

## 1. Introduction

Aconite is a processed product of the lateral root of *Aconitum carmichaelii* Debx. and a plant of the Ranunculaceae family. It is an authentic medicinal material produced in Sichuan. It was first published in “Shennong's Classic of Materia Medica,” which records its spicy, sweetness, hot property, and toxicity [1]. Aconite has a long history of application, and its clinical effect is remarkable. There are 23 proven recipes based on aconite in “Treatise on Febrile and Miscellaneous Diseases” by Zhang Zhongjing, a famous doctor in the Eastern Han Dynasty. Aconite is known as the most important medicine for reviving yang for resuscitation and is respected as one of the “four dimensions of medicine” [2]. The diester alkaloid contained in aconite is its main active ingredient, which can be used for the treatment of heart failure, rheumatic heart disease, coronary heart disease, hypotension, shock, etc. However, aconite is a type of famous toxic Chinese medicine, and the diester alkaloid is a toxic ingredient. This results in higher toxicity to the cardiovascular system, nervous system, digestive system, liver, and kidney [3]. Therefore, it is urgent to use the “double-edged sword” safely and rationally and remove the constraints that affect its clinical application. Based on the bibliometric method, this paper combed the studies on the toxicity of aconite in recent decades and evaluated and analyzed the research hotspots of aconite. In addition, we summarized the toxicity of aconite and related factors.

## 2. Methods

### 2.1. Literature Collection

Relevant studies on the toxicity of aconite were retrieved from CNKI (https://www.cnki.net/), CQVIP (http://www.cqvip.com/), Chinese Biomedical Literature Service System (http://www.sinomed.ac.cn/), and PubMed (https://www.ncbi.nlm.nih.gov/). The retrieval time was from January 1985 to November 2020. The retrieval formula used by each platform is as follows.

The following words are used as keywords or subject terms for literature retrieval in CNKI: “附子 or 附片 or 盐附子 or 白附片 or 黑附子 or 黑顺片 or 黑片 or 顺片 or 熟附子 or 熟片 or 制附子 or 制附片 or 川附子 or 淡附子 or 淡附片 or 炮附片 or 泥附子” with the publication years from 1985 to 2020.

The following words are used as keywords or subject terms for literature retrieval in CQVIP : *M* = (附子 OR 附片 OR 盐附子 OR 白附片 OR 黑附子 OR 黑顺片 OR 黑片 OR 顺片 OR 熟附子 OR 熟片 OR 制附子 OR 制附片 OR 川附子 OR 淡附子 OR 淡附片 OR 炮附片 OR 泥附子) AND (副作用 OR 毒 OR 不良反应 OR 毒效 OR 毒理 OR 毒副作用 OR 不良事件 OR 毒 OR 安全性 OR 中毒).

The following words are used as keywords or subject terms for literature retrieval in Chinese Biomedical Literature Service System: (“附子” [All fields: intelligent] OR “附片” [All fields: intelligent] OR “盐附子” [All fields: intelligent] OR “白附片” [All fields: intelligent] OR “黑附子” [All fields: intelligent] OR “黑顺片” [All fields: intelligent] OR “黑片” [All fields: intelligent] OR “顺片” [All fields: intelligent] OR “熟附子” [All fields: intelligent] OR “熟片” [All fields: intelligent] OR “制附子” [All fields: intelligent] OR “制附片” [All fields: intelligent] OR “川附子” [All fields: intelligent] OR “淡附子” [All fields: intelligent] OR “淡附片” [All fields: intelligent] OR “炮附片” [All fields: intelligent] OR “泥附子” [All fields: intelligent] OR “炮天雄” [All fields: intelligent] OR “制天雄” [All fields: intelligent]) AND (“副作用” [All fields: intelligent] OR “毒” [All fields: intelligent] OR “不良反应” [All fields: intelligent] OR “毒效” [All fields: intelligent] OR “毒理” [All fields: intelligent] OR “毒副作用” [All fields: intelligent] OR “不良事件” [All fields: intelligent] OR “毒” [All fields: intelligent] OR “安全性” [All fields: intelligent] OR “中毒” [All fields: intelligent]).

The following words are used as keywords or subject terms for literature retrieval in PubMed: (Aconitum [MeSH Terms]) OR (Aconite [Title/Abstract]) OR (Aconites [Title/Abstract]) OR (Radix Aconiti [Title/Abstract])) OR (Aconiti, Radix [Title/Abstract]) OR (Aconitus, Radix [Title/Abstract]) OR (Radix Aconitus [Title/Abstract]) OR (Aconitum napellus [Title/Abstract]) OR (Monkshood [Title/Abstract]) OR (Aconitums [Title/Abstract]) AND (poisoning [MeSH Terms]) OR (Side effects [MeSH Terms]) OR (adverse effect [Title/Abstract]) OR (side reaction [Title/Abstract]) OR (toxic effect[Title/Abstract]) OR (toxicity [Title/Abstract]).

### 2.2. Establishment of Database of the Literature of Toxicity of Aconite (DLTA)

After deleting the duplicate studies, 445 studies related to the toxicity of aconite were obtained to form the Database of Literature of Toxicity of Aconite (DLTA).

**Figure 1:**
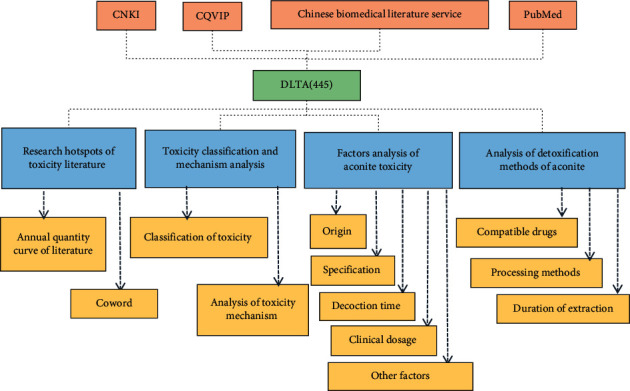
Flow chart of data collection and analysis of studies of aconite toxicity.

We evaluated the research hotspot of toxicity literature, toxicity classification, mechanism analysis, influencing factors of toxicity, and detoxification methods of aconite by the bibliometric method. The collected information included the annual publication of aconite toxicity literature, coword analysis, toxicity classification, toxicity mechanism, aconite varieties, origin, aconite usage, dosage, processing situation, compatible herbs, decoction method, and decoction time. Studies in the DLTA were screened and classified one by one and were structured according to the information collection table. Finally, the extracted data information was classified and analyzed, as shown in Figure 1. Related results can be analyzed and graphed with VOSviewer.

## 3. Results

### 3.1. Research Hotspots of Toxicity Literature of Aconite

#### 3.1.1. Annual Quantity Curve of Literature

**Figure 2:**
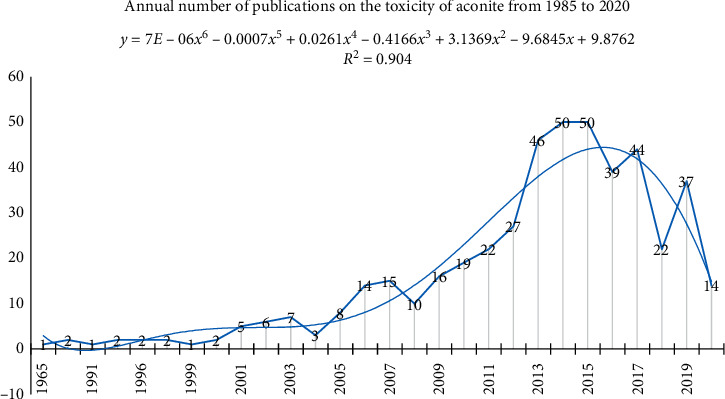
Annual number of publications on the toxicity of aconite from 1985 to 2020.

There were a total of 445 studies related to the toxicity of aconite in the DLTA. The annual number of publications on the toxicity of aconite from 1985 to 2020 was calculated, as shown in Figure 2. 2007 to 2015 is the peak period of the annual number of publications on the toxicity of aconite, and the annual number of publications has shown a downward trend since 2015.

#### 3.1.2. Coword Analysis

**Figure 3:**
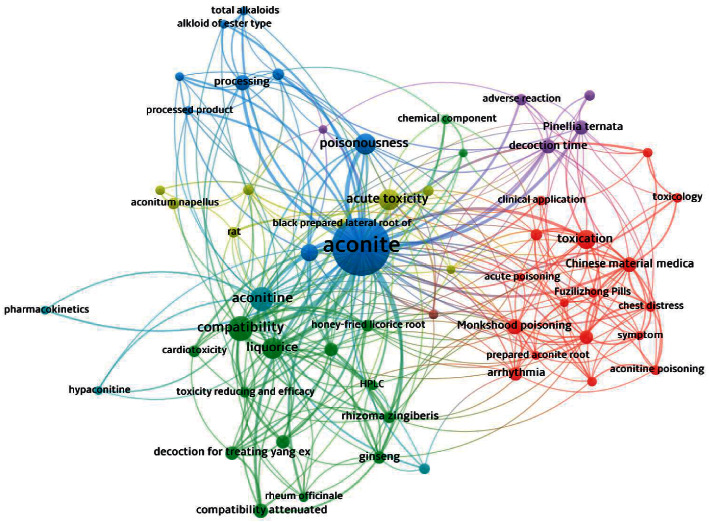
Network visualization in DLTA.

We performed clustering analysis through VOSviewer1.6.15 for coword analysis. Cluster analysis is a multivariate index statistical method that classifies multiple data according to the degree of distance relationship between objects [4]. All studies in DLTA were imported into VOSviewer for coword clustering analysis. They were divided into eight groups with distinguished colors. The size of the node indicates the word frequency, and the number of connections among lines indicates its tightness. As shown in Figure 3, “aconite,” “aconitine,” “cardiomyocyte,” “compatibility,” “licorice,” “poisoning,” “processing,” and “arrhythmia” are the words with the highest frequency. Combined with the coword analysis, the arrhythmia caused by aconite damage to myocardial cells is a common toxic reaction of aconite. Thus, the use of licorice or processing to reduce toxicity is a research hotspot in aconite (Table 1).

### 3.2. Toxicity Classification and Mechanism Analysis

#### 3.2.1. Classification of Toxicity of Aconite

In Table 2, the toxic effects of aconite in DLTA include cardiac damage (including arrhythmia, atrial damage, ventricular toxicity, atrioventricular block, and Al-Syndrome), liver toxicity, renal toxicity, and neurotoxicity [9, 13, 14]. 180 studies mentioned the cardiac damage of aconite. Thus, the main toxic effect of aconite is cardiotoxicity, and arrhythmia is more common in clinical practice.

#### 3.2.2. Analysis of the Toxicity Mechanism of Aconite

A total of 223 studies on the action mechanism of aconite were collected. The toxic action mechanism of aconite was extracted and classified. Then, the statistical analysis was carried out. The toxic action mechanism of aconite that was reported only in one study was eliminated. The classification of its toxic mechanism is shown in Table 3. The toxic reaction mechanisms of aconite reported in many studies are as follows. (1) Aconite has the effect of initial excitement and then inhibition on the central nervous system and peripheral nerves. It mainly acts on the medulla oblongata, causing shock or respiratory depression due to bulbar paralysis, respiratory failure, hypoxic encephalopathy, and death in severe cases [7]. (2) Aconite can excite the vagus nerve, inhibit voltage-dependent sodium channels, increase the sodium ion permeability of nerve cells and myocardial cells, and cause arrhythmia [29]. (3) Aconitum in aconite can inhibit most of the mitochondrial enzymes in the myocardium and cause damage to myocardial cells [30]. (4) Aconite can adjust the activity of L-calcium channels to relatively prolong the repolarization, increase the calcium ion concentration, cause calcium ion overload in myocardial cells, and damage cardiomyocytes [31]. (5) Aconite can excite cholinergic nerves and inhibit cholinesterase activity, thereby inhibiting the heart [31].

From the statistical analysis of studies (Table S1), there are many studies on the action mechanism of aconite toxicity on the heart, with in-depth research and a broader perspective. There are few studies on the action mechanism of aconite toxicity on the liver and kidney. However, these toxic reactions are common in clinical research. If there is no exact description of these toxicity mechanisms, it is impossible to avoid toxic side effects and conduct reasonable treatment.

### 3.3. Factors Analysis of Aconite Toxicity

We analyzed the correlation between aconite toxicity and several factors, such as the origin, the specification, the decoction time, and the dosage of aconite. The bibliometric method was used to analyze the relationship between the origin of aconite, the specifications of medicinal materials, the decoction time, the dosage, and the toxicity of aconite.

#### 3.3.1. The Origin of Aconite

**Figure 4:**
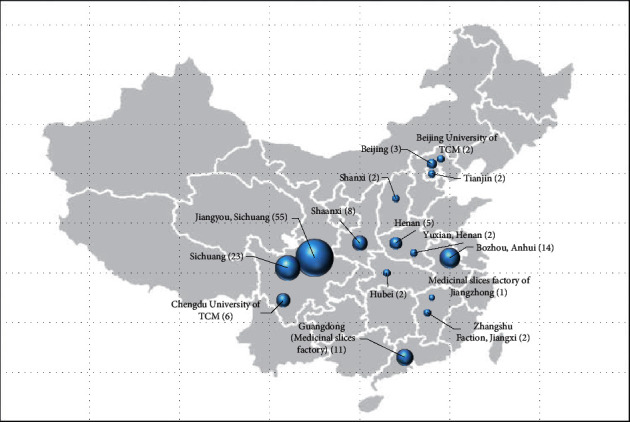
The source of aconite medicinal materials in studies of DLTA (Note: the number in parentheses is the number of studies involved).

The sources of aconite medicinal materials in all studies of DLTA were analyzed, and the classification statistics were performed (Figure 4). Most of the studies reported that the source of aconite is decoction pieces companies or hospitals. It is difficult to trace its source. Therefore, it is impossible to evaluate the relationship between the aconite source and toxicity objectively. In DLTA, 55 studies reported that the aconite is from Jiangyou City, Sichuan. Jiangyou aconite is a well-known authentic medicinal material produced in Sichuan, where it has sophisticated and complicated root-repairing and tipping cultivation techniques [32]. It is a national product of geographic indication. The medicinal ingredients of Jiangyou aconite are more recognized by academia. Therefore, the use of Jiangyou aconite not only ensures clinical safety but also improves the reliability of experimental results.

#### 3.3.2. Aconite Medicinal Material Specifications

**Figure 5:**
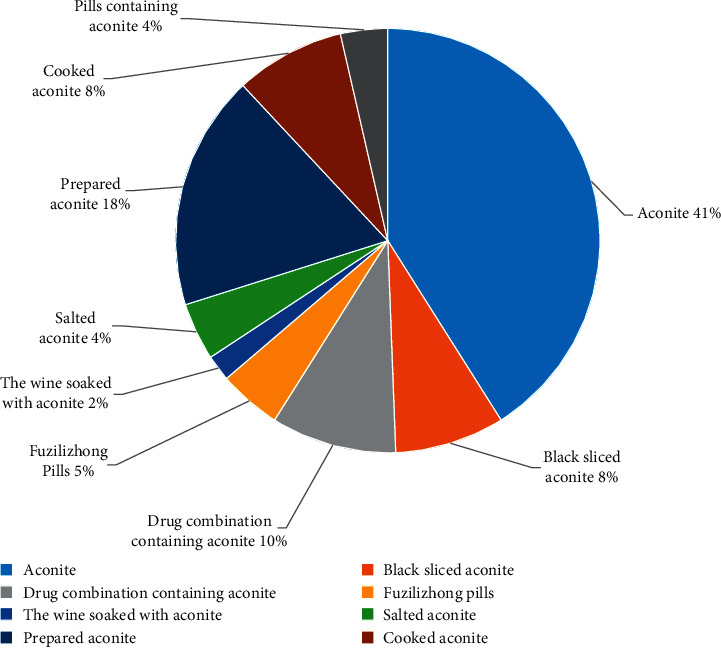
The number of studies of various specifications of aconite in DLTA.

In DLTA, the extracted specifications of aconite medicinal materials include aconite, black aconite/sliced aconite, cooked aconite, prepared aconite, and prescriptions and medicines containing aconite (Table S2). The classification statistics are shown in Table 4 (Figure 5). Huang and Wang [32] summarized the specifications of aconite medicinal materials, including two kinds of aconite and 18 kinds of sliced aconite based on the systematic literature survey, real estate survey, and expert consultation. However, most of the specifications of medicinal materials, such as Linjiang slice, Yangfu slice, Yinfu slice, Liuye slice, and Gugu slice, have gradually disappeared and lost due to various reasons. Therefore, we have not made a statistical analysis on the specifications of medicinal materials with smaller yields.

Literature studies have shown that aconite medicinal materials have many specifications. Most of those specifications are toxic. However, in general, raw aconite has a higher toxicity than other specifications of aconite. Therefore, studies that use “raw aconite or aconite” as the research object are mostly aimed at analyzing its toxicity mechanism or reducing toxicity by compatibility, etc., and there are few clinical studies. The literature with “processed aconite” as the research object involves more clinical research.

#### 3.3.3. Decoction Time of Aconite

**Figure 6:**
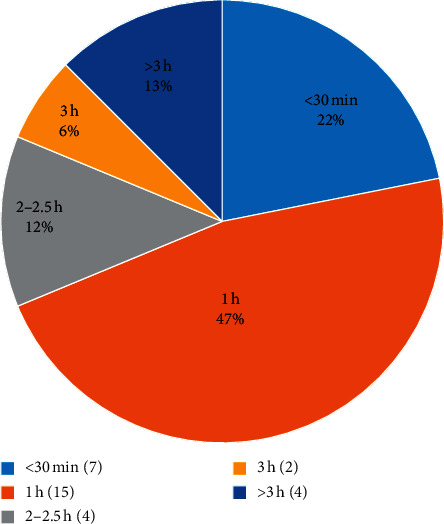
The number of studies of decoction time in the DLTA.

This study analyzed the decoction time of aconite in various studies (Table S3), as shown in Table 5 (Figure 6). The preparation method with decoction time of half an hour or one hour has been widely used. Too short decoction time [47] of aconite or no decoction [48] in the prescription is the common cause of toxicity.

Scholars generally believe that a decoction time of less than 30 minutes is likely to cause clinical toxic events. The decoction time should be extended as the dose of aconite increases. However, most clinicians rely on their own experience about the corresponding relationship between dosage and decoction time, and there is no standard for it. This is one of the reasons for the prone to poisoning reaction when using large doses of aconite [49]. Usually, when the dosage of aconite is less than 30 g, it can be decocted for half an hour. When the dosage is 40∼70 g, it should be decocted for 1 h. If the dosage is more than 70 g, it should be decocted for more than 2 h [50].

#### 3.3.4. Clinical Dosage of Aconite

**Figure 7:**
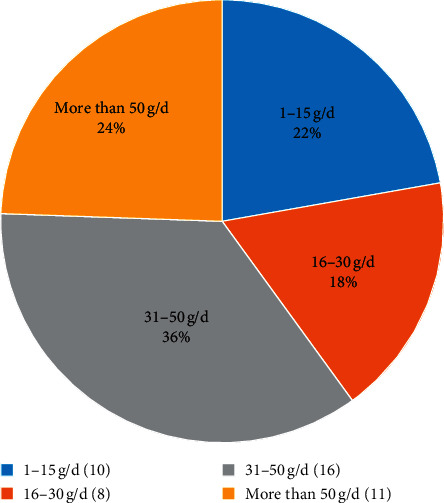
The number of studies on different dosages of aconite in DLTA.

The current textbooks and pharmacopeias stipulate that the common dosage of aconite is 3–15 g/d [51], and aconite is not suitable for long-term use. However, 16 reports mentioned that patients still have poisoning reactions even after reasonably taking aconite and its medicine preparations at normal doses [52], especially the toxicity caused by Aconite Lizhong Pills [38, 53]. The textbooks and pharmacopeias stipulate that the usual amount of aconite is 3–15 g/d [54], which is not suitable for long-term use. However, 16 articles mentioned that patients still had toxic reactions when taking aconite and its medicine preparations at normal doses reasonably [55], especially the toxicity caused by Aconite Lizhong Pills [56, 57]. There are also many cases of overdose or long-term use in clinical practice. Daily doses exceeding 50 g are very common (Figure 7, Table 6). Ultralarge doses of aconite are mostly used to treat intractable diseases such as rheumatoid arthritis and heart block. Although overdose use will increase the probability of aconite poisoning, doctors will mostly reduce toxicity by extending the decoction time and compatibility of medicinal materials. Therefore, the clinical use of aconite does not have strict limits on the dose, and the dose distribution range is relatively wide. There are individual differences in the dose of aconite in clinical application. Even if it is taken according to the instructions or pharmacopeia methods, toxic reactions may still occur. The clinical dose of aconite is positively correlated with toxicity. The use of overdose can also be recognized by doctors in the context of measuring the risk-benefit ratio for some difficult and miscellaneous diseases. The results of the classification are shown in Table S4.

#### 3.3.5. Other Factors

**Figure 8:**
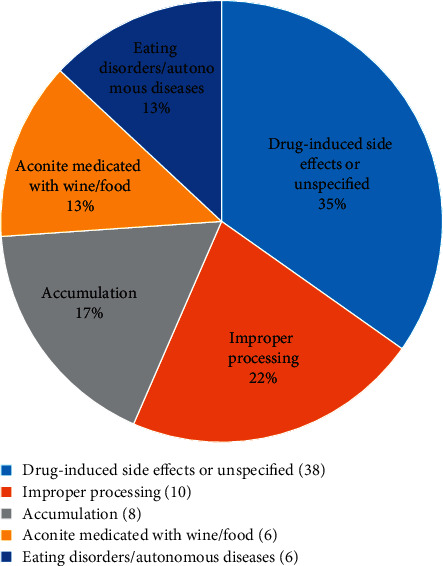
The number of reports on the causes of aconite poisoning in DLTA.

In addition, the factors affecting the toxicity of aconite are shown in Table 7 (Figure 8). (1) Drug-induced side effects or unidentified reason for toxicity: some studies did not clearly explain the cause of poisoning, or the poisoning reaction still occurred without the occurrence of poisoning incentives [63]. (2) Improper processing: for example, the black aconite used excessive bile water in the process [64], causing damage to yang and diarrhea in patients, which are completely unrelated to the symptoms of aconite. “Short decoction time” is also the main cause of poisoning in patients [47]. (3) Drug accumulation [61, 65]: if the patient does not follow the doctor's prescription or take an overlong course of treatment, it may cause poisoning. (4) Aconite medicated with wine or food [62, 66]: six reports in DLTA mentioned that patients took medicinal liquor or medicine containing aconite, which caused toxic reactions. (5) Mistaken eating or self-prescribing [67, 68]: these mostly occur in rural areas and early times. With the improvement of public health awareness, mistaken eating has become rare. The results of the classification are shown in Table S5.

### 3.4. Analysis of Detoxification Methods of Aconite

Through the literature survey, it is found that the commonly used ways to reduce the toxicity of aconite mainly include three methods: drug compatibility, processing, and decoction.

#### 3.4.1. Drug Compatibility

In DLTA, we collected the results and causes of the effects of compatible medicinal materials on the toxicity of aconite and analyzed them statistically, as shown in Table 8. The most commonly compatible drugs with aconite are *Glycyrrhiza uralensis Fisch*, *Zingiber officinale Roscoe*, *Panax ginseng* C. A. Meyer, and *Ephedra sinica* Stapf, etc. The toxicity studies of the compatibility of other drugs with aconite are few. The chemical reaction between aconite and codecocting drugs can reduce toxicity by compatibility, thereby changing the structure of diester alkaloids and converting them into compounds with weaker toxicity [87], or improving enzyme activity to increase the metabolism of aconite [88].

#### 3.4.2. Processing Method

A total of 38 studies on the processing of aconite were collected in this study, and information analysis was performed (Table 9). Studies have shown that because of its high toxicity, raw aconite is rarely taken directly. Most of the processed products of aconite are used clinically. Aconite processing products have many specifications. In recent years, techniques such as puffing and dry heat baking have been introduced in the processing of aconite and have good effects of reducing toxicity [99, 100]. Based on the results of various studies, the toxicity of black sliced aconite, steamed aconite slice, and cooked aconite slice are weaker than that of other processed products of aconite.

In addition, although the safety of aconite is guaranteed, the medicine materials for disease treatment decrease due to overprocessing. Many experimental data supported this view [101, 102]. Only reasonable processing can both reduce the toxicity of raw aconite and give full play to the efficacy. Thus, it is urgent to establish scientific standards of aconite processing. We studied the control of the toxic components of aconite for the safety of the medication and the better curative effect. Therefore, we maintain a balance between toxicity and effect.

#### 3.4.3. Decoction Time

The diester alkaloids in aconite, such as aconitine, hypaconitine, and neoaconitine, are both effective and toxic components [3]. The diester alkaloids can be hydrolyzed by heating or have a lipid exchange reaction with fatty acids to generate fat base and reduce its content, thereby reducing toxicity. Therefore, the pyrolysis reaction caused by aconite heating is also one of the effective ways of aconite detoxification [103].

Through literature surveys (Table 10), it was found that aconitum decoction for more than 60 minutes reached the safe range, and more than 2 hours of decoction may cause the loss of effective components. The toxicity of aconite is closely related to the decocting time, which is a direct factor for the toxicity of aconite.

## 4. Discussion

Based on the bibliometric method, this study evaluated the publication trend and research hotspots of aconite toxicity in the past 35 years. In addition, this paper analyzed the common toxic reactions, toxic mechanisms, factors, and commonly used detoxification methods of aconite. This can provide a useful reference for clinical rational use of aconite and related research on its toxicity.

Since the beginning of 1985, there have been research reports on the toxicity of aconite. The decoction time, dosage, and mode of administration of aconite are the main factors affecting the toxicity of aconite. There are few studies on the effect of the origin of aconite and the specifications of medicinal materials on toxicity, so it is impossible to analyze their relevance. At present, the commonly used methods to reduce the toxicity of aconite include three methods: drug compatibility, concoction, and decoction. The most common drugs compatible with aconite are licorice, dried ginger, ginseng, and ephedra. Black sliced aconite, steamed slices, and fried slices are less toxic than other processed products. Aconite decoction for more than 60 minutes can basically reach the safe range, and more than 2 hours of decoction may cause the loss of active ingredients.

One problem is that the number of studies on the toxicity of aconite has declined in recent years. However, the toxicity of aconite has not been fully revealed. More experimental data and research are needed to confirm and answer: (1) overdose use of aconite (exceeding the pharmacopeia recommendation) is common, but there are few studies on the relationship between quantity and toxicity effect of aconite. When clinical use is caused, there is no uniform reference standard for indication dose, which could easily cause toxic reactions. (2) The reasonable decoction time of aconite is 30 minutes to 1 hour. This has been supported by many studies. However, there are also 2–4 hours of decoction cases in clinical practice, especially in the case of large-dose use. Although long-term decoction reduces the incidence of toxic reactions, does the effect of aconite change after long-term decoction? There are too few research data. The decoction time of large-dose aconite lacks a reference standard. (3) Although there are many studies about the processing and compatibility of aconite, there is a large convergence, the processing method is rarely innovative, and the compatibility research is mostly concentrated on several drugs such as licorice, and the compatibility evaluation with other drugs is less, leading to the lack of precise evidence to guide doctors when prescribing drugs in the clinic, and it is also easy to cause toxic events. (4) More and more reports show that aconite can cause adverse reactions such as kidney and liver damage, but the mechanism of its hepatotoxicity and nephrotoxicity is less. The exact toxicity mechanism needs further study. The researchers should be committed to improve the research of toxicity of aconite, remove the constraints for application, and promote the safe and reasonable use of aconite in the clinic. Researchers should improve various toxicity studies of aconite, remove the constraints that affect its clinical application, and promote the rational use of aconite.

## Figures and Tables

**Table 1:** Top 59 representative keywords in terms of occurrences and total link strength.

No.	Label	Occurrences	Total link strength
1	Aconite	206	326
2	Compatibility	39	95
3	Aconitine	36	65
4	Acute toxicity	29	40
5	Poisonousness	29	46
6	Liquorice	29	81
7	Toxication	23	35
8	Alkaloid	19	41
9	Processing	15	28
10	Monkshood poisoning	15	16
11	Chinese material medica	13	44
12	Pinellia ternata	13	26
13	Decoction time	13	26
14	Compatibility attenuated	13	22
15	Metabonomics	12	26
16	Decoction for treating yang exhaustion	12	19
17	Reduction of toxicity	11	23
18	Arrhythmia	11	15
19	Patient	11	33
20	Ginseng	10	27
21	Rhizoma zingiberis	9	31
22	Adverse reaction	8	13
23	Aconitum napellus	8	6
24	Ester alkaloids	8	18
25	Cardiotoxicity	8	11
26	Honey-fried licorice root	8	24
27	Lateral root slices	8	15
28	Toxicity reducing and efficacy enhancing	7	15
29	Rat	7	10
30	Security	7	12
31	Myocardial cell	7	8
32	Crude lateral root of aconite	7	13
33	Rhizoma typhonii	7	6
34	HPLC	6	12
35	Clinical application	6	8
36	Aconitine poisoning	6	12
37	Chemical component	6	10
38	Eighteen incompatibilities	6	13
39	Rheum officinale	6	14
40	Mouse	6	16
41	Electrocardiograph	6	15
42	Toxicology	6	11
43	Neurovirulence	6	5
44	Chest distress	6	27
45	Fuzi Lizhong pills	6	8
46	Black prepared lateral root of aconite	6	9
47	Compatibility of Chinese medicine	5	8
48	Prepared aconite root	5	14
49	Heart rate	5	17
50	Acute poisoning	5	10
51	Total alkaloids	5	12
52	Hypaconitine	5	6
53	Poisonous components	5	9
54	Processed product	5	12
55	Symptom	5	17
56	Sliced white aconite	5	11
57	Pharmacokinetics	5	5
58	Alkloid of ester type	5	13
59	High-efficiency liquid chromatography	5	14

**Table 2:** Classification of toxicity of aconite.

Toxicity classification	Reference
Cardiac damage	Sheng et al. [5]
Liver toxicity	Hao et al. [6]
Renal toxicity	Hao et al. [6]
Neurotoxicity	Pan and Peng [7]
Gastrointestinal toxicity	Han and Hou [8]
Respiratory system damage	Jiang. [9]
Endocrine dysfunction	Hao et al. [6]
Neurotoxicity of brain	Han et al. [10]
Hematopoietic system damage	Liu. [11]
Embryotoxicity/reproductive organ damage	Xiao et al. [12]

**Table 3:** The classification of the toxicity action mechanism of aconite and the number of studies.

The toxicity action mechanism of aconite	Occurrence	Reference
The effect on various nerve fiber endings and the central nervous system is initial excitement and then inhibition (the effect of the central nervous system may be related to the promotion of the release of *β*-endorphin by aconite)	42	Han et al. [10]
Exciting the vagus nerve reduces the autonomy of the sinus node	40	Li [15]
Inhibiting voltage-dependent sodium channels, increasing the sodium ion permeability of nerve cells and myocardial cells, and causing arrhythmia	27	Sheng et al. [5]
Inhibiting the acid cycle of myocardial tricarboxylic and the acidification of oxidative phosphorylation of the respiratory chain, causing myocardial cell damage and necrosis to release myocardial enzymes	19	Strzelecki et al. [16]
Adjusting L-calcium channel activity to relatively prolong repolarization, increasing calcium ion concentration, and causing calcium ion overload in cardiac myocytes	12	Zhou et al. [17]
Exciting cholinergic nerves, inhibiting cholinesterase activity	11	Wang et al. [18]
Inhibiting the vasomotor center	10	Zhang [19]
Inhibiting the Na-K-ATPase activity of the myocardial cell membrane, leading to a large amount of depletion of myocardial high-energy phosphate bonds, and causing damage to myocardial cells	9	Sun et al. [20]
Causing damage to peripheral nerve	7	Wang [21]
Increasing RyR2 protein expression level	4	Peng [22]
*β*2 adrenergic receptor agonism	4	Lu et al. [23]
Increasing the release of active substances such as prostaglandins and catecholamines	4	Gao and Huang [24]
Promoting the expression of NCX and SERCA2a genes to increase cellular Ca2+ concentration	2	Fu [25]
Causing damage to the DNA of lung fibroblasts	2	Xie [26]
Downregulating gene expression level of Bcl-2 and upregulating gene expression levels of bax	2	Wang et al. [27]
Significantly reducing the mRNA and protein expression levels of PGC-1*α* in cardiomyocytes	2	Zhao et al. [28]

**Table 4:** The occurrence of aconite medicinal material specifications.

Specification	Occurrences	Reference
Aconite	103	Li et al. [33]
Prepared aconite	45	Zhang et al. [34]
Drug combination containing aconite	24	Xu et al. [35]
Black sliced aconite	21	Peng et al. [36]
Cooked aconite	21	Xie et al. [37]
Fuzi Lizhong pills	12	Wang and Liu [38]
Salted aconite	11	Guo et al. [39]
Pills containing aconite	9	Jin et al. [40]
The wine soaked with aconite	5	Wu [41]

**Table 5:** The occurrence of decoction time.

Decoction time	Occurrence	Reference
<30 min	7	Wen et al. [42]
1-2 h	15	Chen et al. [43]
2-2.5 h	4	Luo and Zhang [44]
3 h	2	Fu [45]
>3 h	4	Tan et al. [46]

**Table 6:** The occurrence of dosage.

Dosage	Occurrence	Reference
1–15 g/d (10)	10	Gao and Huang [24]
16–30 g/d (8)	8	Hou et al. [58]
31–50 g/d (16)	16	Zhang et al. [59]
More than 50 g/d (11)	11	Wang and Cai [60]

**Table 7:** The occurrence of other influencing factors.

Other influencing factors	Occurrence	Reference
Drug-induced side effects or unspecified (38)	38	Wang [52]
Improper processing (10)	10	Zhang [19]
Accumulation (8)	8	Wang [61]
Aconite medicated with wine/food (6)	6	Wu [62]
Eating disorders/autonomous diseases (6)	6	Cao [53]

**Table 8:** The effect of compatible drugs on aconite toxicity and its causes.

Compatible drugs	Number of articles	Toxic effect on aconite	Reason	Reference
Licorice	28	Reduce toxicity	① Glycyrrhizic acid and glycyrrhetinic acid in licorice can neutralize with the aconite alkaloids in aconite, and the flavonoids in licorice can also combine with aconitum alkaloids to form a precipitate, both of which can delay or reduce the absorption of toxic alkaloids such as aconitine. ② Glycyrrhizic acid in the gastrointestinal tract can be converted into glycyrrhetinic acid and flavonoids. Licorice flavonoids contain multiple hydroxyl groups, which can combine with alkaloids in aconite to form ester alkaloid precipitation, reduce the content of toxic alkaloids.	Yang et al. [69]
Dried ginger	11	Reduce toxicity	The chemical components in dried ginger can convert the more toxic diester alkaloids in aconite into less toxic ester alkaloids and can antagonize the central inhibitory effect of aconitine, thereby achieving the purpose of detoxification	Yue et al. [70]
Ginseng	8	Reduce toxicity	Ginsenosides in ginseng can increase the SOD activity of cardiomyocytes, reduce MDA content and LDH release rate, and can inhibit the apoptosis of aconite on cardiomyocytes and effectively inhibit its toxic effects.	Wang et al. [27]
Pinellia	6	Toxic increase/decrease	① The compatibility of aconite with qing pinellia, pinellia ginger, and raw pinellia can inhibit the hydrolysis reaction of the alkaloids in aconite, resulting in a significant increase in the content of diester alkaloids. ② The compatibility of aconite and pinellia ternata is attenuated and can make the toxicity more toxic. Large diester alkaloids are transformed into less toxic monoester alkaloids. ③ Compatibility of aconite and pinellia can inhibit CYP1A2 and CYP3A1 enzyme activity, inhibit drug metabolism, and enhance the toxicity.	Huang [71] Jin et al. [72]
Ephedra and Fuzi licorice soup	5	Reduce toxicity	It can significantly reduce the content of diester alkaloids, and the codecocting effect of the three is the best.	Wang and Wan [73]
Ephedra	5	Reduce toxicity	After the two are compatible, the content of monoester alkaloids-benzoyl neoaconitine and benzoyl hypoaconitine is reduced, thereby generating a new ester alkaloid-8-linoleoyl-14- benzoyl hypoaconitine and 8-linoleyl-14-benzoyl aconitine reduces the toxicity.	Pi et al. [74]
Rhubarb	4	Reduce toxicity	During the decoction, the tannins and aconite alkaloids contained in rhubarb produce aconitine salt of tannic acid that is not absorbed by the intestine, thereby reducing the toxicity of aconite, and the content of aconitine decreases as the dose of rhubarb increases. Those are linearly related, and its attenuation effect also increases with the increasing dose of rhubarb.	Wang et al. [75]
Fritillaria Zhejiang/Fritillaria Chuan	4	Toxic increase/decrease	① After codecoction of aconite and fritillaria, the content of aconitine, hypoaconitine, and neoaconitine increased significantly, and the dissolution rate of toxic components of aconitine increased. ② After the compatibility of aconite and fritillaria cirrhosa, the amount of the three diester alkaloids aconitine, mesaconitine, and hypoaconitine was significantly reduced or undetectable, and the toxicity was reduced.	Bian et al. [76] Dong et al. [77]
*Trichosanthes* kirilowii	2	Increase toxicity	The combination of aconite and *Trichosanthes kirilowii* showed serious toxic effects, including promoting heart and kidney inflammation, increasing myocardial fibrosis, and activating *β*2-AR/PKA signal.	Sun et al. [78]
*Astragalus*	2	Reduce toxicity	① Compatible with astragalus can reduce the 6 alkaloids of aconite to varying degrees (benzoyl hypoaconitine BHA, benzoyl neoaconitine BMA, benzoyl aconitine BAC, hypoaconitine HA, new aconitine MA, aconitine AC) plasma concentration. ② Astragalus inhibits the absorption of aconite alkaloids that may be related to the expression of astragalus-induced efflux transporter. ③ Astragalus promotes the clearance of aconite that may be related to the induction of corresponding metabolic enzymes (CYP3A4, CYP3A4, astragalus, CYP1A1, CYP2E1); activity is related.	Liu et al. [79] Zhang et al. [80] Lou et al. [81]
White Peony	1	Reduce toxicity	The diester-type alkaloids in aconite react with the chemical components in the white peony root, so that hypoaconitine, which is not easily hydrolyzed, generates lipid alkaloids. The lipid exchange reaction leads to a decrease in the content of hypoaconitine, thereby achieving attenuation.	Yue et al. [70]
Guizhi	1	Reduce toxicity	The compatibility of Aconite with Guizhi can reduce the total alkaloids and ester alkaloids of aconite, thereby reducing the poisonousness of aconite, and may be able to guide aconite to dispel cold and relieve pain and warm meridians and improve the pulse.	Ye et al. [82]
Cinnamon	1	Unknown	The compatibility of aconite with cinnamon can promote the dissolution of the effective components of aconite and can better guide the aconite to play the role of warming yang and igniting fire.	Ye et al. [82]
Windproof	1	Reduce toxicity	Improve LD50 and TD50	Zhang et al. [83]
Polygala	1	Reduce toxicity	Improve LD50 and TD50	Zhang et al. [83]
Dogwood	1	Reduce toxicity	Enhance the effect of “Wen tongxinyang” of aconite, and reduce its cardiotoxicity.	Jin et al. [84]
Trichosanthin	1	Reduce toxicity	Subacute toxicity experiments in mice show that the toxicity of aconite and *Trichosanthes* is less than that of aconite single decoction.	Yang et al. [85]
Rifampin	1	Reduce toxicity	Rifampicin is a liver drug enzyme inducer, which induces aconite metabolism to accelerate and significantly reduces the acute toxicity of aconite.	Chen et al. [86]
Dry Rehmannia	1	Reduce toxicity	Induces CYP1A2 and CYP3A4 enzyme activity, increases CYP450 enzyme content, accelerates the metabolism of toxic components of aconite, and achieves aconite attenuation [53].	Li et al. [33]

**Table 9:** Determination results and comparison of specifications and toxic content of processing products of aconite.

Aconite processing specifications	The determination results	Conclusions	Reference
Black sliced aconite, baifupian, mud aconite	The LD_50_ of alcohol extracts of black sliced aconite, baifupian, and mud aconite is 49.853 g kg^−1^, 42.550 g kg^−1^, and 22.169 g kg^−1^, respectively	Toxicity: black sliced aconite < baifupian < mud aconite	Xie et al. [89]
Bafupian, black sliced aconite, salted aconite	The LD_50_ of baifupian and black sliced aconite is 20.529 g kg^−1^. Salted aconite is more toxic with LD_50_ of 11.301 g kg^−1^	Toxicity: black sliced aconite = baifupian < salted aconite	Chai et al. [90]
Shengfupian, baifupian, Heifupian, Paofupian	The LD_50_ of the water extract and alcohol extract of shengfupian is 22.4 g kg^−1^ and 13.2 g kg^−1^, respectively; the maximum tolerable dosages of baifupian, heifupian, and paofupian are 533 g, 666 g, and 266 g, respectively	Toxicity: water extract of shengfu pian < alcohol extract of shengfu pian; heifupian < baifupian < paofupian	Zhou et al. [91]
Aconite puffed decoction pieces	The content of diester-type alkaloids of aconite: 0.13952% for raw aconite, 0.03771% for black sliced aconite, 0.05896% for baifupian, and 0.05024% for aconite puffed decoction pieces	Toxicity: black sliced aconite < aconite puffed decoction pieces < baifupian < raw aconite	Cheng. [92]
dry heat baking, moist heat baking	After processing, the content of diester alkaloids of aconite significantly decreases or disappears, and the content of monoester alkaloids increases significantly	Reducing toxicity	Tang et al. [93]
High voltage sliced aconite	The content of monoester alkaloids is higher, and the content of toxic components of diester alkaloids significantly decreases. High-pressure aconite tablets have the advantages of high efficiency and low toxicity	Active ingredients: high voltage sliced aconite > high temperature sliced aconite, microwave sliced aconite, black sliced aconite	Jia et al. [94]
Fried sliced aconite	Compared with other processed aconite products, fried sliced aconite has a lower content of diester alkaloids, the highest content of monoester alkaloids, and the lowest content of ephedra alkaloids	Toxicity: fried sliced aconite < black sliced aconite, danfupian, baifupian, paofupian	Qiu [95]
Raw aconite, prepared aconite	Raw aconite has a better effect than processed aconite on Rhubarb aconite decoction	Rhubarb aconite decoction using raw aconite has a better therapeutic effect and is safe to use	Guo [96]
Black sliced aconite, fried sliced aconite, steamed sliced aconite	The maximum tolerated amount of fried sliced aconite is 170 g kg^−1^, that of steamed sliced aconite is 268 g kg^−1^, and the LD_50_ of Heishunian is 138.13 g	Toxicity: steamed sliced aconite < fried sliced aconite < black sliced aconite	Zhang et al. [97]
Danba preparation	Among different salted products, aconite with 40% and 45% concentration of danba preparation has lower toxicity, and the former one also has an obvious neuroprotective effect	40% danba should be used for salted aconite	Liu [98]

**Table 10:** The effect of decocting time on aconite toxicity.

Type	Detection indicator	Conclusion	Reference
Acute toxicity test in mice	Biochemical indicators, mortality, adverse reactions, etc.	The toxicity of aconite decoction for 60 minutes is relatively low, and the pharmacological activity is the strongest; when the decoction exceeds 105 minutes, the animals in each group behave normally without death	Kao and Zhang [104]
Clinical safety experiment	Nausea, vomiting, dizziness, salivation, and other adverse reactions	The normal dose decoction time should be controlled within 1-2 h; but if the dose is more than 200 g, an additional 1 h decoction time should be added	Liang et al. [105]
HPLC content determination	Aconitine, neoaconitine, hypoaconitine	Both the 0 min and 30 min water decoctions of aconite are toxic, but the 60 min toxicity is not significant, and it can basically be defined as nontoxic	Sun et al. [106]
HPLC and UV methods	Neoaconitine, hypoaconitine, aconitine, benzoyl neoaconitine, benzoyl hypoaconitine, benzoyl aconitine	After 0.5 h of decoction, the content of diester alkaloids basically disappeared. After 1 h of decoction, the content of monoester alkaloids and total alkaloids reached the maximum	Gong et al. [107]
HPLC content determination	Aconitine, hypoaconitine, neoaconitine, benzoyl neoaconitine, benzoyl hypoaconitine, benzoyl aconitine	After decoction of black sliced aconite for 3.5 hours, the content of monoester alkaloids gradually disappeared	Lin et al. [108]
HPLC content determination	13 kinds of alkaloids including aconitine, neoaconitine and hypoaconitine	After decoction of shengfu tablets for 2–4 hours, the content of diester alkaloids is already very low, which can ensure the safety of clinical medication	Zhang et al. [109]
HPLC content determination	Neoaconitine, hypoaconitine, aconitine, benzoyl neoaconitine, benzoyl hypoaconitine, benzoyl aconitine	The diester alkaloids in raw aconite are extremely unstable in water decoction. Hypoaconitine was detected within 0.5 h of decoction	Chen et al. [110]
HPLC content determination	Aconitum alkaloids	After decocting the aconite in water for 30 minutes, the content of aconitine and hypoaconitine became 10.5% and 41.9% of the peak value, respectively, and aconitine was completely undetectable; after the aconite microwave heating for 150s, the content of aconitine, neoaconitine, and hypoaconitine was 59.2%, 41.4%, and 86.6% of the peak value, respectively	Sui et al. [111]
HPLC and UV methods	Total alkaloids, ester alkaloids, polysaccharide components, diester alkaloid components	The best decocting time is within 1 hour	Yu et al. [112]

## Data Availability

The datasets generated during and/or analyzed during the current study are available from CNKI (https://www.cnki.net/), CQVIP (http://www.cqvip.com/), Chinese Biomedical Literature Service System (http://www.sinomed.ac.cn/), and PubMed (https://www.ncbi.nlm.nih.gov/).

## Supplementary Materials

Supplementary Materials Table S1: the classification of the toxicity action mechanism of aconite and the number of studies. Figure S2: the number of studies of various specifications of aconite in DLTA. Table S3: the number of studies of decoction time in the DLTA. Table S4: the number of studies on different dosages of aconite in DLTA. Table S5: the number of reports on the causes of aconite poisoning in DLTA.
