# Supplementary material for: Systematic Evaluation of Toxicity of Aconite Based on Bibliometric Method
Source: Evid Based Complement Alternat Med. 2021 Aug 2;2021:5514281. doi: 10.1155/2021/5514281 (PMC8363442; doi:10.1155/2021/5514281)
Supplement: Supplementary Materials — Table S1: the classification of the toxicity action mechanism of aconite and the number of studies. Figure S2: the number of studies of various specifications of aconite in DLTA. Table S3: the number of studies of decoction time in the DLTA. Table S4: the number of studies on different dosages of aconite in DLTA. Table S5: the number of reports on the causes of aconite poisoning in DLTA. [file 5514281.f1.docx]

**TABLE S1 The classification of the toxicity action mechanism of aconite and the number of literatures.**

| **The toxicity action mechanism of aconite** | Num | ARTICLES |  |  |
| --- | --- | --- | --- | --- |
| The effect on various nerve fiber endings and the central nervous system is initial excitement and then inhibition (the effect of the central nervous system may be related to the promotion of the release of β-endorphin by aconite) | 1 | 张媛. 硕士, 成都中医药大学 (2008). |  |  |
|  | 2 | 杨媛. 硕士, 北京中医药大学 (2008). |  |  |
|  | 3 | 韩屾, 吕雷, 王汉蓉, 陈波, 彭成, 王莉,岑小波. 3种乌头类中药在大鼠体内外的神经毒性. 华西药学杂志 22, 286-288 (2007). |  |  |
|  | 4 | 张宽民. 附子中毒38例临床分析. 内科急危重症杂志 11(2005). |  |  |
|  | 5 | 王良馥,陈自力. 综合抢救重度附子中毒7例. 中国中医药信息杂志 12, 86-87 (2005). |  |  |
|  | 6 | 何少峰. 小剂量附子中毒致恶性心律失常、心搏骤停1例. 中国煤炭工业医学杂志 8(2005). |  |  |
|  | 7 | 唐春荣. 附子中毒1例. 中国中药杂志 27(2002). |  |  |
|  | 8 | 王仁轼. 常规剂量附片中毒致恶性心律失常1例. 四川医学 22, 509 (2001). |  |  |
|  | 9 | 王建凯,刘国良. 附子理中丸致中毒反应2例报道. 实用中医药杂志 17, 43 (2001). |  |  |
|  | 10 | 罗显田. 附子中毒致恶性心律失常、心脏骤停1例报道. 海军医学杂志 (2000). |  |  |
|  | 11 | 何忠文. 附子,川乌,草乌中毒12例辨析. 江西中医学院学报 12(2000). |  |  |
|  | 12 | 齐敏. 服附子理中丸致中毒1例报告. 内蒙古中医药 0(1994). |  |  |
|  | 13 | 周长绵,黄健. 抢救急性附子中毒8例临床报告. 新中医, 39-40 (1991). |  |  |
|  | 14 | 张化为. 硕士, 陕西中医药大学;陕西中医学院 (2009). |  |  |
|  | 15 | 徐彪. 硕士, 河北医科大学 (2010). |  |  |
|  | 16 | 文永兰, 李亚玲,李俊. 白附片中毒一例. 实用药物与临床 12, 435 (2009). |  |  |
|  | 17 | 秦婉玲,施恒. 炙甘草汤治疗附子中毒一例. in 江西省中西医结合学会第九次活血化瘀学术研讨会活血化瘀临床应用新进展培训班论文集 (中国江西南昌, 2011). |  |  |
|  | 18 | 侯胜福, 张湘兰,林春. 中药附子急性中毒1例. 临床合理用药杂志 04, 73-73 (2011). |  |  |
|  | 19 | 李勋. 一例误服附子中毒晕厥的抢救护理体会. 健康必读（下旬刊）, 256 (2012). |  |  |
|  | 20 | 梁文坚, 王清海, 李典鸿,杨跃娜. 附子中毒致恶性心律失常1例报道. 按摩与康复医学, 119-119,120 (2014). |  |  |
|  | 21 | 李梦婷, et al. 基于斑马鱼研究附子脂溶性总生物碱的神经毒性. 中药药理与临床 35, 63-66 (2019). |  |  |
|  | 22 | 张宽民 ,任文生,王彩玲.附子中毒38例临床分析[J].内科急危重症杂志,2005(04):187-188. |  |  |
|  | 23 | 李红专. 黄连、栀子、附子、仙茅对甲状腺及胰腺内分泌功能的影响及机制研究[D].山东中医药大学,2014. |  |  |
|  | 24 | 武志娟. 附子理中汤治疗腹泻型肠易激综合征的临床与实验研究[D].广州中医药大学,2013. |  |  |
|  | 25 | 李志勇. 附子成分次乌头碱心脏毒性及中毒机制研究[D].北京中医药大学,2008. |  |  |
|  | 26 | 张建军. 乌头类中药雄性大鼠生殖毒性体内外试验研究[D].四川大学,2007. |  |  |
|  | 27 | 肖凯. 乌头类中药大鼠胚胎发育毒性体内外试验研究[D].四川大学,2006. |  |  |
|  | 28 | 朱林平. 阳虚、阴虚模型大鼠应用附子毒性对比及其机制的初步研究[D].天津中医学院,2005. |  |  |
|  | 29 | 徐红萌. 乳铁蛋白和附子对神经病理性疼痛大鼠的镇痛作用[D].河北医科大学,2005. |  |  |
|  | 30 | 张宏. 附子、川乌、草乌、草乌叶时间、剂量与药效的相关性研究[D].成都中医药大学,2005. |  |  |
|  | 31 | 何健,吴萍,董宇,高蕊.附子不良反应分析及应用网络药理学对其产生心脏毒性的机制预测[J].中国中药杂志,2019,44(05):1010-1018. |  |  |
|  | 32 | 方成鑫. 附子水溶性化学成分及其心脏毒性研究[D].成都中医药大学,2018. |  |  |
|  | 33 | 刘红梅. 胆巴炮制对附子化学成分及神经毒性影响[D].成都中医药大学,2018. |  |  |
|  | 34 | 潘校琦,彭成.附子神经毒性研究进展[J].世界中医药,2017,12(11):2551-2554+2562. |  |  |
|  | 35 | 赵欢,杨巧芳,李梦茜.附子毒性研究进展[J].河北中医,2017,39(05):774-777. |  |  |
|  | 36 | 赵志浩,张定堃,吴明权,李春雨,曹丽娟,张萍,刘翠哲,王伽伯,肖小河.基于大鼠室性早搏心脏毒性的附子质量生物评价方法研究[J].中国中药杂志,2016,41(20):3814-3820. |  |  |
|  | 37 | 熊海霞. 基于“有故无殒”理论的病/证因素对附子及其复方毒性和药效影响的研究[D].北京中医药大学,2015. |  |  |
|  | 38 | 沈玉巧. 附子现代炮制品的急性毒性及药效学评价研究[D].广州中医药大学,2015. |  |  |
|  | 39 | 陈荣昌,孙桂波,张强,叶祖光,孙晓波.附子毒性研究进展[J].中国中药杂志,2013,38(08):1126-1129. |  |  |
|  | 40 | 温瑞卿. 基于生物毒价检测和化学成分分析的毒性中药附子质量评控研究[D].成都中医药大学,2013. |  |  |
|  | 41 | 谢晓芳. 附子心脏毒作用机制研究[D].成都中医药大学,2012. |  |  |
|  | 42 | 孙凤姣. 基于β2-AR/PKA信号探讨附子瓜蒌配伍对压力超负荷大鼠炎症反应和心肌纤维化的增毒作用. 中国中药杂志 0(2019). |  |  |
| Exciting the vagus nerve reduces the autonomy of sinus node | 1 | 杨媛. 硕士, 北京中医药大学 (2008). |  |  |
|  | 2 | 韩屾, 吕雷, 王汉蓉, 陈波, 彭成, 王莉,岑小波. 3种乌头类中药在大鼠体内外的神经毒性. 华西药学杂志 22, 286-288 (2007). |  |  |
|  | 3 | 吴玲毓. 附片中毒致心律失常3例报告. 中国社区医师(综合版), 68 (2006). |  |  |
|  | 4 | 刘筱蔼, 吴伟康,梁天文. 煎煮方法和药物配伍对附子毒性的影响. 医药世界, 98-101 (2006). |  |  |
|  | 5 | 张宽民. 附子中毒38例临床分析. 内科急危重症杂志 11(2005). |  |  |
|  | 6 | 宋友湘. 附子中毒致恶性心律失常分析. 国际医药卫生导报 (2005). |  |  |
|  | 7 | 张爱萍. 制附片中毒致心律失常及四相完全性右束支传导阻滞1例. 现代中西医结合杂志 12(2003). |  |  |
|  | 8 | 陈玉秋. 附子理中丸中毒致严重休克心律失常l例. 吉林医学信息 019, 42 (2002). |  |  |
|  | 9 | 沈桢巍, 冯兰英,徐红. 乌头碱中毒致严重心律失常二例. 中华心律失常学杂志 5, 370-370 (2001). |  |  |
|  | 10 | 乐永宏. 急性附子中毒伴胆碱酯酶活性抑制1例. 中国误诊学杂志 1, 154 (2001). |  |  |
|  | 11 | 何忠文. 附子,川乌,草乌中毒12例辨析. 江西中医学院学报 12(2000). |  |  |
|  | 12 | 王宁波,张学臻. 附子中毒致严重心律失常1例. 医学文选 18, 1006 (1999). |  |  |
|  | 13 | 周长绵,黄健. 抢救急性附子中毒8例临床报告. 新中医, 39-40 (1991). |  |  |
|  | 14 | 郭华林,郭杰. 附子与半夏中药急性中毒的救治体会. 中国急救医学 31, 272-273 (2011). |  |  |
|  | 15 | 党中文. 65例乌头碱中毒临床分析. 云南医药 33, 410-411 (2012). |  |  |
|  | 16 | 张庆辉. 附子理中丸中毒致心律失常1例. 临床荟萃 25, 2082 (2010). |  |  |
|  | 17 | 刘玉庆,戴雁彦. 附子中毒1例. in 第二届长城国际中西医结合心脏病论坛论文集 168-170 (北京, 2011). |  |  |
|  | 18 | 蔡吉芬, 杨云贵, 周丽琼, 罗亚坤,董家能. 附子中毒156例临床分析. 昆明医学院学报 32, 131-132 (2011). |  |  |
|  | 19 | 洪彩萍. 附子中毒病人的抢救护理体会. 健康必读（中旬刊）, 89 (2011). |  |  |
|  | 20 | 梁小坤. 急性附子中毒23例临床分析. 临床医药实践B版 19, 745-746 (2010). |  |  |
|  | 21 | 王义臣,王尽美. 口服中药附子煎剂致严重中毒1例. 中国医学创新 6, 50 (2009). |  |  |
|  | 22 | 刘莉. 制附片中毒致室性心律失常2例. 中外健康文摘 09, 289-290 (2012). |  |  |
|  | 23 | 刘智, 张大方, 曲晓波, 皮子凤, 王立岩, 张志仁,王奕琛. 炮制对附子减毒变化及氯仿致颤作用的比较研究. 吉林中医药 31, 469-470,474 (2011). |  |  |
|  | 24 | 叶金枝,何青霞. 乌头中毒致多形室性心动过速1例. 实用心电学杂志 18, 431-431 (2009). |  |  |
|  | 25 | 李勋. 一例误服附子中毒晕厥的抢救护理体会. 健康必读（下旬刊）, 256 (2012). |  |  |
|  | 26 | 马斌智. 超常规剂量苍耳子中毒致室速1例报告. 青海医药杂志 (2013). |  |  |
|  | 27 | 许少辉, 曾艳,刘玉英. 附子中毒的急救护理及预防. 海南医学 24, 1859-1861 (2013). |  |  |
|  | 28 | 蒋志青. 乌头、附子引起急性中毒反应1例临床探讨. 黑龙江中医药 43, 24 (2014). |  |  |
|  | 29 | 李志勇, et al. 四逆汤配伍环境下的附子“效-毒网络”交集调控研究. 中国中药杂志 40, 733-738 (2015). |  |  |
|  | 30 | 黄梦婷, et al. 服用附片中毒引起Ⅲ度房室传导阻滞及凝血功能异常1例报道. 现代医药卫生 33, 3210-3211 (2017). |  |  |
|  | 31 | 杨齐,陈静. 阿托品联合利多卡因治疗乌头碱中毒引起的室性心律失常疗效分析. 云南医药 38, 357-358 (2017). |  |  |
|  | 32 | 张蕾, 付一程, 李宏林, 刘巨海,彭欣. 薏苡仁配伍附子对大鼠心脏毒性影响的实验研究. 时珍国医国药 28, 92-95 (2017). |  |  |
|  | 33 | 张春喜, 杨宏恬,杨春洪. 八例重度乌头碱中毒致快速室性心律失常救治体会. 云南医药 39, 273,279 (2018). |  |  |
|  | 34 | 邹俊驹, 陆雅婷, 欧阳建军, 赵国荣,贺又舜. 附子理中汤对失血性低血压大鼠心率及心肌酶的影响. 湖南中医杂志 34, 161-164 |  |  |
|  | 35 | 盛开, 田强强, 汪浩浩,赵启明. 附子总碱对大鼠心功能及离体心脏的损害作用及其机制. 基因组学与应用生物学 38, 4709-4714 |  |  |
|  | 36 | 李朝容,彭文静. 服用常规剂量附片中毒致二度房室传导阻滞1例. 基层医学论坛 24, 1467-1468 (2020). |  |  |
|  | 37 | 余葱葱. 配伍环境改变调控附子毒性的机制研究[D].成都中医药大学,2009. |  |  |
|  | 38 | 李伶. 附子及其主要毒性成分对大鼠毒性的代谢组学研究[D].中国人民解放军军事医学科学院,2007. |  |  |
|  | 39 | 陈学习. 附子对病证动物模型基础毒性作用的实验研究[D].成都中医药大学,2006. |  |  |
|  | 40 | 潘德林, 张翅, 唐莉, 朱雅宁,樊官伟. 参附强心颗粒中附片减毒增效工艺研究. 中国中医急症 29, 95-99 (2020). |  |  |
| Inhibiting voltage-dependent sodium channels, increasing the sodium ion permeability of nerve cells and myocardial cells, and causing arrhythmia | 1 | 徐彪. 硕士, 河北医科大学 (2010). |  |  |
|  | 2 | 刘玉庆,戴雁彦. 附子中毒1例. in 第二届长城国际中西医结合心脏病论坛论文集 168-170 (北京, 2011). |  |  |
|  | 3 | 刘智, 张大方, 曲晓波, 皮子凤, 王立岩, 张志仁,王奕琛. 炮制对附子减毒变化及氯仿致颤作用的比较研究. 吉林中医药 31, 469-470,474 (2011). |  |  |
|  | 4 | 叶金枝,何青霞. 乌头中毒致多形室性心动过速1例. 实用心电学杂志 18, 431-431 (2009). |  |  |
|  | 5 | Strzelecki, A., Pichon, N., Gaulier, J.M., Amiel, J.B., Champy, P.,Clavel, M. Acute toxic herbal intake in a suicide attempt and fatal refractory ventricular arrhythmia. Basic Clin Pharmacol Toxicol 107, 698-699 (2010). |  |  |
|  | 6 | 李莎, et al. 口服附子炮制品导致潜在性心律失常的机理研究. in 中国毒理学会中药与天然药物毒理专业委员会第一次(2016年)学术交流大会论文集 |  |  |
|  | 7 | 刘筱蔼, 吴伟康,梁天文. 煎煮方法和药物配伍对附子毒性的影响. 医药世界, 98-101 (2006). |  |  |
|  | 8 | 徐彪. 硕士, 河北医科大学 (2010). |  |  |
|  | 9 | 李志勇, et al. 四逆汤配伍环境下的附子“效-毒网络”交集调控研究. 中国中药杂志 40, 733-738 (2015). |  |  |
|  | 10 | 李莎, et al. 口服附子炮制品导致潜在性心律失常的机理研究. in 中国毒理学会中药与天然药物毒理专业委员会第一次(2016年)学术交流大会论文集 55-56 (天津, 2016). |  |  |
|  | 11 | 赵佳伟. 硕士, 安徽医科大学 (2016). |  |  |
|  | 12 | 龚小红. 博士, 成都中医药大学 (2017). |  |  |
|  | 13 | 黄梦婷, et al. 服用附片中毒引起Ⅲ度房室传导阻滞及凝血功能异常1例报道. 现代医药卫生 33, 3210-3211 (2017). |  |  |
|  | 14 | 滕培颍. 博士, 广州中医药大学 (2017). |  |  |
|  | 15 | 汪戎锦, 丁茹,刘忠英. 基于三重四级杆串联质谱技术研究甘草附子汤中乌头类生物碱的吸收转运机制. in 第三届全国质谱分析学术报告会论文集 316-316 (厦门, 2017). |  |  |
|  | 16 | 杨齐,陈静. 阿托品联合利多卡因治疗乌头碱中毒引起的室性心律失常疗效分析. 云南医药 38, 357-358 (2017). |  |  |
|  | 17 | 马增春, et al. 乌头碱引发心肌细胞钙离子超载的作用机制. in 2018第八届药物毒理学年会——创新技术、评估风险、保障安全 1 (中国江苏南京, 2018). |  |  |
|  | 18 | 彭文鹏. 附子所致中毒性心肌炎一例. 饮食保健 5, 82-83 (2018). |  |  |
|  | 19 | 张春喜, 杨宏恬,杨春洪. 八例重度乌头碱中毒致快速室性心律失常救治体会. 云南医药 39, 273,279 (2018). |  |  |
|  | 20 | 邹俊驹, 陆雅婷, 欧阳建军, 赵国荣,贺又舜. 附子理中汤对失血性低血压大鼠心率及心肌酶的影响. 湖南中医杂志 34, 161-164 (2018). |  |  |
|  | 21 | 马增春, et al. 乌头碱对心肌细胞线粒体毒效转换作用研究. in 第九届药物毒理学年会——新时代·新技术·新策略·新健康 2 (中国湖北武汉, 2019). |  |  |
|  | 22 | 盛开, 田强强, 汪浩浩,赵启明. 附子总碱对大鼠心功能及离体心脏的损害作用及其机制. 基因组学与应用生物学 38, 4709-4714 (2019). |  |  |
|  | 23 | 李浩然. 硕士, 华中科技大学 (2016). |  |  |
|  | 24 | 李莹, et al. 四逆汤中甘草减附子之毒的代谢组学研究. 中国中药杂志 41, 1523-1529 (2016). |  |  |
|  | 25 | 李朝容,彭文静. 服用常规剂量附片中毒致二度房室传导阻滞1例. 基层医学论坛 24, 1467-1468 (2020). |  |  |
|  | 26 | 张硕峰. 附子中三种双酯型生物碱的心脏毒效关系及甘草苷的干预作用[D].北京中医药大学,2007. |  |  |
|  | 27 | 潘德林, 张翅, 唐莉, 朱雅宁,樊官伟. 参附强心颗粒中附片减毒增效工艺研究. 中国中医急症 29, 95-99 (2020). |  |  |
| Inhibiting the acid cycle of myocardial tricarboxylic and the acidification of oxidative phosphorylation of the respiratory chain, causing myocardial cell damage and necrosis to release myocardial enzymes | 1 | 李瑞煜, 张定堃, 韩雪, 林俊芝,杨明. 基于代谢组学方法的制附片煎煮过程成分变化规律及煎煮时限探讨. 中草药 47, 38-45 (2016). |  | |
|  | 2 | 李莹. 博士, 成都中医药大学 (2013). |  | |
|  | 3 | 李莹, et al. 基于MI-RI大鼠心肌细胞代谢组学研究四逆汤中附子配伍甘草解毒增效机制. 中国中药杂志 39, 3166-3171 (2014). |  | |
|  | 4 | 都姣娇, 苏红宁,齐淑静. 附子主要成分对大鼠心肌细胞的毒性分析. 首都食品与医药 22, 93-94 (2015). |  | |
|  | 5 | 熊静悦, 牟道华, 唐大轩,谭正怀. 白附子急性毒性作用研究. 四川生理科学杂志 32, 101-103 (2010). |  | |
|  | 6 | 周子渝, 熊永爱, 黄勤挽,杨明. 附子不同炮制品及其部位急性毒性研究. 成都中医药大学学报 35, 63-65 (2012). |  | |
|  | 7 | 孙世晓, et al. 生附子、生半夏配伍后急性毒性的实验研究. 中医药信息 28, 104-106 (2011). |  |  |
|  | 8 | 马增春, et al. 参附配伍对附子毒性减低作用. in 中国毒理学会第六届全国毒理学大会论文集 103-104 (广州, 2013). |  |  |
|  | 9 | 肖勇. 硕士, 中南大学 (2014). |  |  |
|  | 10 | 肖勇, et al. 参附注射液配伍对乌头碱诱发心律失常的减毒研究. 中药药理与临床 29, 12-15 (2013). |  |  |
|  | 11 | 李志勇, et al. 四逆汤配伍环境下的附子“效-毒网络”交集调控研究. 中国中药杂志 40, 733-738 (2015). |  |  |
|  | 12 | 黄梦婷, et al. 服用附片中毒引起Ⅲ度房室传导阻滞及凝血功能异常1例报道. 现代医药卫生 33, 3210-3211 (2017). |  |  |
|  | 13 | 徐彪. 硕士, 河北医科大学 (2010). |  |  |
|  | 14 | 赵佳伟. 硕士, 安徽医科大学 (2016). |  |  |
|  | 15 | 李浩然. 硕士, 华中科技大学 (2016). |  |  |
|  | 16 | 都姣娇, 苏红宁,齐淑静. 附子主要成分对大鼠心肌细胞的毒性分析. 首都食品与医药 22, 93-94 (2015). |  |  |
|  | 17 | 朱林平. 阳虚、阴虚模型大鼠应用附子毒性对比及其机制的初步研究[D].天津中医学院,2005. |  |  |
|  | 18 | 杨齐,陈静. 阿托品联合利多卡因治疗乌头碱中毒引起的室性心律失常疗效分析. 云南医药 38, 357-358 (2017). |  |  |
|  | 19 | 陈彦旭, et al. 利福平对附子急性毒性物质诱导降解的研究. 中国现代中药 21, 786-790 (2019). |  |  |
| Adjusting L-calcium channel activity to relatively prolong repolarization, increasing calcium ion concentration, and causing calcium ion overload in cardiac myocytes | 1 | 赵佳伟, 何家乐, 马增春, 梁乾德, 王宇光, 谭洪玲, 肖成荣, 汤响林,高月. 附子对H9c2心肌细胞系线粒体的毒性作用机制. 中国药理学与毒理学杂志, 816-824 (2015). |  |  |
|  | 2 | 周天梅, 杨洁红, 万海同, 张宇燕,周惠芬. 附子甘草主要成分配伍对乌头碱致大鼠传代心肌细胞损伤的保护作用. 北京中医药大学学报 37, 22-26,后插23 (2014). |  |  |
|  | 3 | 李志勇, et al. 四逆汤配伍环境下的附子“效-毒网络”交集调控研究. 中国中药杂志 40, 733-738 (2015). |  |  |
|  | 4 | 李莎, et al. 口服附子炮制品导致潜在性心律失常的机理研究. in 中国毒理学会中药与天然药物毒理专业委员会第一次(2016年)学术交流大会论文集 55-56 (天津, 2016). |  |  |
|  | 5 | 马增春,高月. 参附配伍减毒作用的系统研究. in 2016年第六届全国药物毒理学年会 1 (中国重庆, 2016). |  |  |
|  | 6 | 黄梦婷, et al. 服用附片中毒引起Ⅲ度房室传导阻滞及凝血功能异常1例报道. 现代医药卫生 33, 3210-3211 (2017). |  |  |
|  | 7 | 汪戎锦, 丁茹,刘忠英. 基于三重四级杆串联质谱技术研究甘草附子汤中乌头类生物碱的吸收转运机制. in 第三届全国质谱分析学术报告会论文集 316-316 (厦门, 2017). |  |  |
|  | 8 | 马增春, et al. 乌头碱引发心肌细胞钙离子超载的作用机制. in 2018第八届药物毒理学年会——创新技术、评估风险、保障安全 1 (中国江苏南京, 2018). |  |  |
|  | 9 | 彭文鹏. 附子所致中毒性心肌炎一例. 饮食保健 5, 82-83 (2018). |  |  |
|  | 10 | 邹俊驹, 陆雅婷, 欧阳建军, 赵国荣,贺又舜. 附子理中汤对失血性低血压大鼠心率及心肌酶的影响. 湖南中医杂志 34, 161-164 (2018). |  |  |
|  | 11 | 马增春, et al. 乌头碱对心肌细胞线粒体毒效转换作用研究. in 第九届药物毒理学年会——新时代·新技术·新策略·新健康 2 (中国湖北武汉, 2019). |  |  |
|  | 12 | 李朝容,彭文静. 服用常规剂量附片中毒致二度房室传导阻滞1例. 基层医学论坛 24, 1467-1468 (2020). |  |  |
| Exciting cholinergic nerves, inhibiting cholinesterase activity | 1 | 侯胜福, 张湘兰,林春. 中药附子急性中毒1例. 临床合理用药杂志 04, 73-73 (2011). |  |  |
|  | 2 | 徐彪. 硕士, 河北医科大学 (2010). |  |  |
|  | 3 | 王瑞, 展晓日,乔延江. 附子总碱提取物的急性毒性实验研究. 中国实验方剂学杂志 15, 102-103 (2009). |  |  |
|  | 4 | 钟梅芳. 麻黄附子甘草汤的不同配伍方式对其毒性成分的影响分析. 实用中医内科杂志 26, 68-69 (2012). |  |  |
|  | 5 | 刘智, et al. 炮制对附子减毒变化及氯仿致颤作用的比较研究. 吉林中医药 31, 469-470,474 (2011). |  |  |
|  | 6 | 余葱葱. 博士, 成都中医药大学 (2009). |  |  |
|  | 7 | 张晓晨,郑清阁,杨菁华,容蓉,杨勇.附子C_(19)二萜生物碱结构及活性研究进展[J].中草药,2020,51(02):531-541. |  |  |
|  | 8 | 李梦婷,张慧琼,文瑞琪,向郁森,彭成,谢晓芳.基于斑马鱼研究附子脂溶性总生物碱的神经毒性[J].中药药理与临床,2019,35(06):63-66. |  |  |
|  | 9 | 徐新华. 附子的毒性及双酯型生物碱成分多剂量药物动力学研究[D].广州中医药大学,2013. |  |  |
|  | 10 | 章鹏. 附子安全应用及不同煎煮方法所得附子复方煎煮液对小鼠的急性毒性试验[D].北京中医药大学,2010. |  |  |
|  | 11 | 张蕾, 付一程, 李宏林, 刘巨海,彭欣. 薏苡仁配伍附子对大鼠心脏毒性影响的实验研究. 时珍国医国药 28, 92-95 (2017). |  |  |
| Inhibiting the vasomotor center | 1 | 韩屾, 吕雷, 王汉蓉, 陈波, 彭成, 王莉,岑小波. 3种乌头类中药在大鼠体内外的神经毒性. 华西药学杂志 22, 286-288 (2007). |  |  |
|  | 2 | 张爱萍. 制附片中毒致心律失常及四相完全性右束支传导阻滞1例. 现代中西医结合杂志 12(2003). |  |  |
|  | 3 | 刘莉. 制附片中毒致室性心律失常2例. 中外健康文摘 09, 289-290 (2012). |  |  |
|  | 4 | 侯胜福, 张湘兰,林春. 中药附子急性中毒1例. 临床合理用药杂志 04, 73-73 (2011). |  |  |
|  | 5 | 尚翠香, 乔丽红, 王晓红,袁欢. 综合措施救治附子中毒致心跳呼吸骤停1例. 陕西中医 30, 1396 (2009). |  |  |
|  | 6 | 黄梦婷, et al. 服用附片中毒引起Ⅲ度房室传导阻滞及凝血功能异常1例报道. 现代医药卫生 33, 3210-3211 (2017). |  |  |
|  | 7 | 杨齐,陈静. 阿托品联合利多卡因治疗乌头碱中毒引起的室性心律失常疗效分析. 云南医药 38, 357-358 (2017). |  |  |
|  | 8 | 张春喜, 杨宏恬,杨春洪. 八例重度乌头碱中毒致快速室性心律失常救治体会. 云南医药 39, 273,279 (2018). |  |  |
|  | 9 | 陈学习. 附子对病证动物模型基础毒性作用的实验研究[D].成都中医药大学,2006. |  |  |
|  | 10 | 陈彦旭, et al. 利福平对附子急性毒性物质诱导降解的研究. 中国现代中药 21, 786-790 (2019). |  |  |
| Inhibiting the Na-K-ATPase activity of myocardial cell membrane, leading to a large amount of depletion of myocardial high-energy phosphate bonds, and causing damage to myocardial cells | 1 | 韩屾, 吕雷, 王汉蓉, 陈波, 彭成, 王莉,岑小波. 3种乌头类中药在大鼠体内外的神经毒性. 华西药学杂志 22, 286-288 (2007). |  |  |
|  | 2 | 胡一冰. 博士, 成都中医药大学 (2005). |  |  |
|  | 3 | 谭勇. 博士, 中国中医科学院 (2009). |  |  |
|  | 4 | 周天梅, 杨洁红, 万海同, 张宇燕,周惠芬. 附子甘草主要成分配伍对乌头碱致大鼠传代心肌细胞损伤的保护作用. 北京中医药大学学报 37, 22-26,后插23 (2014). |  |  |
|  | 5 | 孙燕,王雨,刘京华,王斯维,王小莹.附子双酯型二萜生物碱对代谢酶的影响及心肌毒性研究进展[J].天津中医药大学学报,2018,37(04):274-279. |  |  |
|  | 6 | 熊海霞. 基于“有故无殒”理论的病/证因素对附子及其复方毒性和药效影响的研究[D].北京中医药大学,2015. |  |  |
|  | 7 | 沈玉巧. 附子现代炮制品的急性毒性及药效学评价研究[D].广州中医药大学,2015. |  |  |
|  | 8 | 李志勇. 附子成分次乌头碱心脏毒性及中毒机制研究[D].北京中医药大学,2008. |  |  |
|  | 9 | 黄梦婷, et al. 服用附片中毒引起Ⅲ度房室传导阻滞及凝血功能异常1例报道. 现代医药卫生 33, 3210-3211 (2017). |  |  |
| Causing damage to peripheral nerve | 1 | 杨媛. 硕士, 北京中医药大学 (2008). |  |  |
|  | 2 | 罗显田. 附子中毒致恶性心律失常、心脏骤停1例报道. 海军医学杂志 (2000). |  |  |
|  | 3 | 蒋志青. 乌头、附子引起急性中毒反应1例临床探讨. 黑龙江中医药 43, 24 (2014). |  |  |
|  | 4 | 杨媛. 硕士, 北京中医药大学 (2008). |  |  |
|  | 5 | 李朝容,彭文静. 服用常规剂量附片中毒致二度房室传导阻滞1例. 基层医学论坛 24, 1467-1468 (2020). |  |  |
|  | 6 | 赵佳伟. 硕士, 安徽医科大学 (2016). |  |  |
|  | 7 | 王贝贝. 附子对糖尿病周围神经病变的活性与机制探讨及乌头碱类成分的识别[D].北京中医药大学,2016. |  |  |
| Increasing RyR2 protein expression level | 1 | 马增春, et al. 乌头碱引发心肌细胞钙离子超载的作用机制. in 2018第八届药物毒理学年会——创新技术、评估风险、保障安全 1 (中国江苏南京, 2018). |  |  |
|  | 2 | 彭文鹏. 附子所致中毒性心肌炎一例. 饮食保健 5, 82-83 (2018). |  |  |
|  | 3 | 马增春, et al. 乌头碱对心肌细胞线粒体毒效转换作用研究. in 第九届药物毒理学年会——新时代·新技术·新策略·新健康 2 (中国湖北武汉, 2019). |  |  |
|  | 4 | 李浩然. 硕士, 华中科技大学 (2016). |  |  |
| β2 adrenergic receptor agonism | 1 | 孙凤姣. 基于β2-AR/PKA信号探讨附子瓜蒌配伍对压力超负荷大鼠炎症反应和心肌纤维化的增毒作用. 中国中药杂志 0(2019). |  |  |
|  | 2 | 邓晓红,黄建华,董竞成.附子药理作用的分子机制研究进展[J].江西中医药大学学报,2018,30(01):121-124. |  |  |
|  | 3 | 卢志强,张艳军,庄朋伟,张金保,周会芳,陈卓,孙萌,徐利满.附子对急性心力衰竭大鼠血流动力学影响及其机制研究[J].中草药,2015,46(21):3223-3227. |  |  |
|  | 4 | 李志勇. 附子成分次乌头碱心脏毒性及中毒机制研究[D].北京中医药大学,2008. |  |  |
| Increasing the release of active substances such as prostaglandins and catecholamines | 1 | 高凯,黄春林. 附子提取物的大鼠生殖毒性研究. 中国现代药物应用, 20-21 (2014). |  |  |
|  | 2 | 张为良. 附子中毒导致周围神经病变1例. 世界中医药 0(2016). |  |  |
|  | 3 | 黄梦婷, et al. 服用附片中毒引起Ⅲ度房室传导阻滞及凝血功能异常1例报道. 现代医药卫生 33, 3210-3211 (2017). |  |  |
|  | 4 | 李浩然. 硕士, 华中科技大学 (2016). |  |  |
| Promoting the expression of NCX and SERCA2a genes to increase cellular Ca^2+^ concentration | 1 | 付敏. 乌头碱致心律失常的细胞分子机制的实验研究[D].北京中医药大学,2007. |  |  |
|  | 2 | 赵佳伟. 硕士, 安徽医科大学 (2016). |  |  |
| Causing damage to the DNA of lung fibroblasts | 1 | 谢晓芳. 附子心脏毒作用机制研究[D].成都中医药大学,2012. |  |  |
|  | 2 | 王金勇. 硕士, 四川大学 (2007). |  |  |
| Down-regulating gene expression level of Bcl-2 and up-regulating gene expression levels of Bax | 1 | 马增春, 谭洪玲, 肖成荣, 王宇光, 梁乾德, 汤响林,高月. 参附配伍减毒增效作用的系统研究. 345-347 (北京, 2014). |  |  |
|  | 2 | 王晓丽, 李丽静, 李玉梅, 李超英,张大方. 附子与人参不同配伍对心肌细胞的减毒作用. 中国实验方剂学杂志 21, 153-158 (2015). |  |  |
| Significantly reducing the mRNA and protein expression levels of PGC-1α in cardiomyocytes | 1 | 黄光耀. 基于PXR-CYP3A通路对附子不同提取方式下毒性及机制的研究[D].安徽医科大学,2019. |  |  |
|  | 2 | 赵佳伟,何家乐,马增春,梁乾德,王宇光,谭洪玲,肖成荣,汤响林,高月.附子对H9c2心肌细胞系线粒体的毒性作用机制[J].中国药理学与毒理学杂志,2015,29(05):816-824. |  |  |

**Figure S2 The number of literatures of various specifications of aconite in DLTA**

| specifications | NUM | articles | |
| --- | --- | --- | --- |
| aconite | 1 | 王良馥,陈自力. 综合抢救重度附子中毒7例. 中国中医药信息杂志 12, 86-87 (2005). | |
|  | 2 | 张风雷, 李立纪, 吴荣祖,刘培儒. 附子、附片及煎煮液化学成分比较实验及毒性研究. 云南中医中药杂志 25, 29-31 (2004). | |
|  | 3 | 游敏生. 附子中毒1例报告. 中华临床医药杂志（北京） 4(2003). | |
|  | 4 | 唐春荣. 附子中毒1例. 中国中药杂志 27(2002). | |
|  | 5 | 罗显田. 附子中毒致恶性心律失常、心脏骤停1例报道. 海军医学杂志 (2000). | |
|  | 6 | 王宁波,张学臻. 附子中毒致严重心律失常1例. 医学文选 18, 1006 (1999). | |
|  | 7 | 王雅娜. 中西医结合抢救乌头类中药中毒5例. 中国中西医结合杂志 0(1994). | |
|  | 8 | 徐彪. 硕士, 河北医科大学 (2010). | |
|  | 9 | 谢力群. 成功抢救1例中药附子中毒致多器官衰竭的护理体会. 医学信息（上旬刊） 23, 2172-2173 (2010). | |
|  | 10 | 王义臣,王尽美. 口服中药附子煎剂致严重中毒1例. 中国医学创新 6, 50 (2009). | |
|  | 11 | 秦婉玲,施恒. 炙甘草汤治疗附子中毒一例. in 江西省中西医结合学会第九次活血化瘀学术研讨会活血化瘀临床应用新进展培训班论文集 (中国江西南昌, 2011). | |
|  | 12 | 叶金枝,何青霞. 乌头中毒致多形室性心动过速1例. 实用心电学杂志 18, 431-431 (2009). | |
|  | 13 | 尚翠香, 乔丽红, 王晓红,袁欢. 综合措施救治附子中毒致心跳呼吸骤停1例. 陕西中医 30, 1396 (2009). | |
|  | 14 | 田家敏. 经方治疗急腹症验案3则. 中国中医急症 24(2015). | |
|  | 15 | 彭文鹏. 附子所致中毒性心肌炎一例. 饮食保健 5, 82-83 (2018). | |
|  | 16 | 张丽红, 施青青,孙婧. 乌头碱中毒疑似急性冠脉综合征1例. 武警医学 30, 992-993 (2019). | |
|  | 17 | 刘慧. 附子汤治疗轻中度膝骨关节炎寒湿痹阻证临床研究. 临床研究 25, 67-68 (2017). | |
|  | 18 | 白雪, 康晓慧, 杨随庄, 袁小红,周强. 附子生物碱脱毒前后对小菜蛾室内毒力活性比较研究. 湖南师范大学自然科学学报 39, 29-34 (2016). | |
|  | 19 | 白雪. 硕士, 西南科技大学 (2016). | |
|  | 20 | 李秋红, 单万亭, 鞠爱霞,赵娇. 基于肝脏药物代谢酶研究附子配伍干地黄的减毒机制. 中国中药杂志 45, 3961-3966 (2020). | |
|  | 21 | 李梦婷, et al. 基于斑马鱼研究附子脂溶性总生物碱的神经毒性. 中药药理与临床 35, 63-66 (2019). | |
|  | 22 | 季苗苗, et al. 不同培养基条件下黄芪和附子及其配伍对Caco-2细胞毒性的影响. 时珍国医国药 30, 261-264 (2019). | |
|  | 23 | 黄光耀. 硕士, 安徽医科大学 (2019). | |
|  | 24 | 段小花, et al. 不同剂量和煎煮时间的附子煎剂对小鼠非特异性免疫和毒性的影响. 时珍国医国药 30, 345-347 (2019). | |
|  | 25 | 丁兴杰, et al. 附子多糖纯化工艺的优化及其毒性. 中成药 41, 2737-2740 (2019). | |
|  | 26 | 从娇娇, 张雷, 覃晓慧, 张峻颖,吴春勇. 附子毒性生物碱的肝脏摄取机制. 医药导报 38, 711-717 (2019). | |
|  | 27 | 陈彦旭, et al. 利福平对附子急性毒性物质诱导降解的研究. 中国现代中药 21, 786-790 (2019). | |
|  | 28 | 刘红梅. 硕士, 成都中医药大学 (2018). | |
|  | 29 | 李攀, 韩佳, 张世鹏,张帆. 4种川乌类药材急性毒性的差异性研究. 药学研究 37, 562-564 (2018). | |
|  | 30 | 黄光耀, 马增春, 高杉,高月. 附子水提与醇提物对AC16细胞的毒性作用的比较. in 2018第八届药物毒理学年会——创新技术、评估风险、保障安全 2 (中国江苏南京, 2018). | |
|  | 31 | 方成鑫. 硕士, 成都中医药大学 (2018). | |
|  | 32 | 梁效铭. 硕士, 广州中医药大学 (2016). | |
|  | 33 | 梁碧彦, 黄辉霞, 李秀凤,王秀丽. 不同产地生附子同剂量的破格救心汤结合西医常规治疗对难治性心衰的心功能影响. 世界中医药 11, 989-992 (2016). | |
|  | 34 | 李莎, et al. 口服附子炮制品导致潜在性心律失常的机理研究. in 中国毒理学会中药与天然药物毒理专业委员会第一次(2016年)学术交流大会论文集 55-56 (天津, 2016). | |
|  | 35 | 褚雪镭, 杨兴言, 甄蓉蓉, 王彦芳,秦林. “附子走而不守”性能表征的实验研究. 中医药信息 33, 5-7 (2016). | |
|  | 36 | 陈荣. 硕士, 广州中医药大学 (2016). | |
|  | 37 | 柴宁. 附子不同炮制品对麻黄细辛附子汤煎剂质量的影响. 中国处方药 17, 43-44 (2019). | |
|  | 38 | 曾欢. 甘草饮片与甘草配方颗粒在四逆汤中减毒作用比较. 广东药科大学学报 36(2020). | |
|  | 39 | 曹丽梅. 硕士, 成都中医药大学 (2017). | |
|  | 40 | 李成皇. 乌头碱中毒致心律失常1例报告. 中国保健营养（下旬刊） 23, 1166-1166 (2013). | |
|  | 41 | 蒋志青. 乌头、附子引起急性中毒反应1例临床探讨. 黑龙江中医药 43, 24 (2014). | |
|  | 42 | 久服附子中毒案. 上海中医药杂志 48, 32 (2014). | |
|  | 43 | 李彦文. 基于四逆汤配伍环境的附子"效-毒网络"交集调控与减毒规律研究. | |
|  | 44 | 梁文坚, 王清海, 李典鸿,杨跃娜. 附子中毒致恶性心律失常1例报道. 按摩与康复医学, 119-119,120 (2014). | |
|  | 45 | 林华, 方莉, 龚又明,邓广海. 附子高温烘制工艺的正交试验追加法优选. 时珍国医国药 25, 1382-1385 (2014). | |
|  | 46 | 李志生. 综合措施救治半夏与附子急性中毒随机平行对照研究. 实用中医内科杂志 27, 17-18 (2013). | |
|  | 47 | 程芬. 硕士, 西南交通大学 (2013). | |
|  | 48 | 何家乐, et al. 基于代谢组学技术研究参附配伍对大鼠心脏毒性的影响. 中国中药杂志 40, 2743-2747 (2015). | |
|  | 49 | 刘甜甜. 硕士, 成都中医药大学 (2014). | |
|  | 50 | 都姣娇, 苏红宁,齐淑静. 附子主要成分对大鼠心肌细胞的毒性分析. 首都食品与医药 22, 93-94 (2015). | |
|  | 51 | 程显怡, et al. 附子与瓜蒌不同配比急性毒性比较. 西部中医药 26, 13-15 (2013). | |
|  | 52 | 蔡亚梅, 娄子洋, 朱臻宇,柴逸峰. 附子心脏毒性的脂质轮廓分析. in 《中国药学杂志》岛津杯第十一届全国药物分析优秀论文评选交流会论文集 91-95 (南京, 2013). | |
|  | 53 | 高凯,黄春林. 附子提取物的大鼠生殖毒性研究. 中国现代药物应用, 20-21 (2014). | |
|  | 54 | 冯群, 李晓宇, 栾永福, 孙赛男,孙蓉. 附子水提物单次给药对小鼠心脏“量-时-毒”关系研究. 中国中药杂志 40, 927-932 (2015). | |
|  | 55 | 梁佳佳, et al. 附子炮制及与大黄配伍后酯型生物碱的含量变化研究. 世界科学技术-中医药现代化, 38-44 (2014). | |
|  | 56 | 李晓宇, 栾永福,孙蓉. 附子对脾肾阳虚型心衰大鼠的效-毒关联评价. 中国药物警戒, 449-453 (2015). | |
|  | 57 | 李晓骄阳, 栾永福,孙蓉. 附子不同组分对正常小鼠的急性毒性实验比较研究. 中国药物警戒 10, 583-587 (2013). | |
|  | 58 | 梁碧彦, 钟伟湘, 李尚, 李秀凤,王秀丽. 附子不良反应与煎煮时间及剂量的关系. 中国临床新医学, 864-866 (2015). | |
|  | 59 | 代小娇. 硕士, 西南医科大学;泸州医学院 (2013). | |
|  | 60 | 考玉萍,张化为. 大剂量食用附子安全性和有效性研究. 陕西中医 34, 478-480 (2013). | |
|  | 61 | 林彦君, et al. HPLC-Q-TOF/MS指纹技术对比分析附子配伍炙甘草前后化学组分变化. 中草药 45, 1556-1560 (2014). | |
|  | 62 | 陈彦琳, et al. 2010-2011年中医药行业科研专项"附子等中药炮制方法传承与规范化应用研究"项目研究成果的反思与建议. in 2015年全国中药炮制学术年会暨炮制分会换届选举会议论文集 5-5 (大连, 2015). | |
|  | 63 | 李贞景, et al. 4种有毒中药对秀丽隐杆线虫致死率和产卵数的影响. 毒理学杂志 27(2013). | |
|  | 64 | 蓝娟, et al. 炙甘草配伍对附子水煎液中乌头类生物碱的含量和离体肠吸收的影响. 中国药科大学学报 43, 430-434 (2012). | |
|  | 65 | 陈华英, et al. 药酶诱导剂对生附子急性毒性的影响. 辽宁中医药大学学报 12, 30-32 (2010). | |
|  | 66 | 刘智, et al. 炮制对附子减毒变化及氯仿致颤作用的比较研究. 吉林中医药 31, 469-470,474 (2011). | |
|  | 67 | 侯胜福, 张湘兰,林春. 中药附子急性中毒1例. 临床合理用药杂志 04, 73-73 (2011). | |
|  | 68 | 梁小坤. 急性附子中毒23例临床分析. 临床医药实践B版 19, 745-746 (2010). | |
|  | 69 | 李永哲,秦孝智. 附子中毒致严重心律失常1例. 医学理论与实践 23, 760 (2010). | |
|  | 70 | 曹昌雪. 附子中毒1例报道. 实用中医药杂志 28, 705 (2012). | |
|  | 71 | 刘春叶, et al. 附子在炮制中主要生物碱含量变化的研究. 时珍国医国药 23, 960-962 (2012). | |
|  | 72 | 柴玉爽, et al. 附子乌头草乌及其炮制品的毒效比较. 世界科学技术-中医药现代化 13, 847-851 (2011). | |
|  | 73 | 李启艳, 朱日然, 张学顺, 程明,黄超. 附子及其炮制品中生物碱类成分的ESI-MSn研究. 中国实验方剂学杂志 17, 90-93 (2011). | |
|  | 74 | 林娟, 徐君,黄秋云. 大剂量应用附子务必要久煎. 当代医学 18, 36-37 (2012). | |
|  | 75 | 白锦栋. 长期大剂量使用附子一例. 神经药理学报 (1989). | |
|  | 76 | 乐永宏. 急性附子中毒伴胆碱酯酶活性抑制1例. 中国误诊学杂志 1, 154 (2001). | |
|  | 77 | 廖显春. 乌头碱中毒致非阵发性室性心动过速1例报告. 岭南急诊医学杂志 6, 232-233 (2001). | |
|  | 78 | 李蕙君. 附子中毒致严重心律失常2例. 中国实用内科杂志 23(2003). | |
|  | 79 | 陈建宗, 黄晨, 高建苑, 田季雨,奚淑芳. 中药附子中毒引起心律失常10例. 药物流行病学杂志 13, 223-224 (2004). | |
|  | 80 | 郭效建, 刘安述,楮体云. 半夏、附子配伍中毒2例报告. 山东医药 44, 75 (2004). | |
|  | 81 | 陈理书, 张云鹏,杨悦娅. 临床应用附子78例探析. 辽宁中医杂志 32, 348-349 (2005). | |
|  | 82 | 何少峰. 小剂量附子中毒致恶性心律失常、心搏骤停1例. 中国煤炭工业医学杂志 8(2005). | |
|  | 83 | 白海燕, 马建列,陈毅仁. 附子浸渍液对花卉蚜虫的毒力测定及药效试验. 中国农学通报 22, 323-325 (2006). | |
|  | 84 | 边宝林, 司南, 王宏洁, 杨健,何希荣. 附子单煎以及与浙贝母合煎后乌头碱、次乌头碱、新乌头碱等有毒成分的含量变化研究. 中国实验方剂学杂志 12, 9-10 (2006). | |
|  | 85 | 陈学习. 博士, 成都中医药大学 (2006). | |
|  | 86 | 刘筱蔼, 吴伟康,梁天文. 煎煮方法和药物配伍对附子毒性的影响. 医药世界, 98-101 (2006). | |
|  | 87 | 陈金月, 周芳,黄世优. 大剂量使用附子的安全性研究. 亚太传统医药 4, 37-39 (2008). | |
|  | 88 | 梁汝圣, 徐宗佩, 任永丽,张增瑞. 附子毒性辨证研究. 吉林中医药 28, 526-527 (2008). | |
|  | 89 | 时英菊, 石晓华,陈会娟. 生附子中毒急救1例护理体会. 河北中医 33, 1731 (2011). | |
|  | 90 | 1. 李浩然. 硕士, 华中科技大学 (2016). | |
|  | 91 | 2. 李冰洁, et al. 从蛋白质自组装的角度探析甘草附子配伍减毒机制. 中国中药杂志 40, 661-666 (2015). | |
|  | 92 | 3. 刘海学, et al. 不同药性辅料炮制附子对其毒性和强心成分的影响. in 中华中医药学会中药炮制分会2009年学术研讨会论文集 | |
|  | 93 | 4. 韩屾. 硕士, 四川大学 (2007). | |
|  | 94 | 5. 李伶. 硕士, 解放军军事医学科学院;中国人民解放军军事医学科学院 (2007). | |
|  | 95 | 6. 洪波,仇永清. 附子中双酯型乌头碱类成分水解减毒机理的密度泛函理论研究. 分子科学学报 24, 216-219 (2008). | |
|  | 96 | 王征, 武雪, 刘建利, 唐志书,宋忠兴. 川乌和附子的毒性与热性的细胞实验研究. 中华中医药学刊 34, 582-584,后插584 (2016). | |
|  | 97 | 皮子凤, 越皓, 宋凤瑞, 刘志强,刘淑莹. 附子与不同药味药材配伍后乌头碱类生物碱的电喷雾质谱研究. 中草药 39, 1474-1477 (2008). | |
|  | 98 | 丘小惠,何洁. 煎煮时间及甘草配伍剂量对附子中酯型生物碱含量的影响. 时珍国医国药 18, 3015-3017 (2007). | |
|  | 99 | 张梅, 苏筱琳, 雨田,彭成. 五种中药与附子配伍前后有效成分含量的变化. 世界科学技术-中医药现代化 8, 27-29 (2006). | |
|  | 100 | 王勇, 刘志强, 宋凤瑞,刘淑莹. 附子配伍原则的电喷雾质谱研究. 药学学报 38, 451-454 (2003). | |
|  | 101 | 裴妙荣, 王世民,李晶. 四逆汤中甘草对附子解毒作用的相关性分析. 中国中药杂志, 50-52,65 (1996). | |
|  | 102 | 徐姗珺. 甘草与附子配伍减毒的有效成分及作用环节研究. 中成药 28(2006). | |
|  | 103 | 章津铭, 傅超美, 秦素红, 李莹,张臻. LC-MS/MS比较研究附子配伍甘草对大鼠体内次乌头碱药动学影响. 世界科学技术·中医药现代化 13(2011). | |
| Black sliced aconite root | 1 | | 王云. 黑附子及配伍甘草、干姜对小鼠致死毒性随机平行对照研究. 28, 68-70 (2014). |
|  | 2 | | 彭慧, 王妍妍, 张盛, 葛韬, 彭代银,陈卫东. 黑顺片不同组分对正常小鼠的急性毒性. 中成药 40, 1842-1844 (2018). |
|  | 3 | | 王云. 黑附子及配伍甘草、干姜对小鼠致死毒性随机平行对照研究. 28, 68-70 (2014). |
|  | 4 | | 侯新莲,刘杰,周鑫,蔡帮军,彭成,周勤梅.泡胆和漂洗炮制环节对黑顺片质量影响研究[J].时珍国医国药,2020,31(07):1626-1628. |
|  | 5 | | 王晓雅,缪璐琳,熊亮,郭力,彭成,周勤梅.不同产地黑顺片醇提物对斑马鱼毒性和心率的影响[J].中华中医药学刊,2021,39(01):172-174+292. |
|  | 6 | | 熊秋韵,张慧琼,谢晓芳,刘怡,熊亮,邓青秀,彭成.附子不同炮制品对风湿寒痹类风湿关节炎大鼠的急性毒性试验研究[J].中药药理与临床,2020,36(02):149-158. |
|  | 7 | | 张秀,黄伟,赵科,向菊芳,刘琪,曹方引,周雪,谭正怀.不同炮制工艺对附片毒性及功效的影响[J].中药药理与临床,2019,35(01):103-107. |
|  | 8 | | 彭慧,王妍妍,张盛,葛韬,彭代银,陈卫东.黑顺片不同组分对正常小鼠的急性毒性[J].中成药,2018,40(08):1842-1844. |
|  | 9 | | 胡静远. 中药附子炮制机理的研究[D].西南科技大学,2018. |
|  | 10 | | 彭慧. 茯苓配伍黑顺片减毒增效作用研究[D].安徽中医药大学,2018. |
|  | 11 | | 肖建平,陈健民.附子炮制及使用的体会[J].海峡药学,2017,29(01):36-37. |
|  | 12 | | 唐恬,赵婷,赵晖,陈振振.附子及其炮制品对大鼠心脏毒性的影响[J].中国中医急症,2016,25(12):2286-2289+2315. |
|  | 13 | | 陈炯. 制川乌、黑顺片质量考察及物质基础差异性研究[D].北京中医药大学,2016. |
|  | 14 | | 贾雪岩,林华,沈玉巧,邓广海.附子新型炮制品中乌头类生物碱测定及其强心作用研究[J].药物评价研究,2016,39(02):224-229. |
|  | 15 | | 谭茂兰. 黑顺片炮制过程中成分、热性、药效和毒性的变化研究[D].成都中医药大学,2016. |
|  | 16 | | 宋玉琴,张雪,董艳红,代良萍,彭成,谢晓芳.附子不同炮制品灌胃Beagle犬的急性毒性研究[J].世界科学技术-中医药现代化,2015,17(07):1432-1437. |
|  | 17 | 沈玉巧,林华,邓广海,贾雪岩.不同炮制工艺附子煎煮液的急性毒性探讨[J].中国实验方剂学杂志,2015,21(12):121-124. | |
|  | 18 | 汪云伟. 基于化学成分及气味的附子（黑顺片）产地加工炮制过程研究[D].成都中医药大学,2015. | |
|  | 19 | 沈玉巧. 附子现代炮制品的急性毒性及药效学评价研究[D].广州中医药大学,2015. | |
|  | 20 | 罗学凤.浅谈黑顺片的合理应用[J].内蒙古中医药,2014,33(11):48+3. | |
|  | 21 | 龚又明,方莉,林华,邓广海,冯倩茹.附子不同部位及其炮制品生物碱的含量比较[J].北方药学,2013,10(08):6-8. | |
| Drug combination contaning aconite | 1 | 徐建东, 王洪泉, 张文英,吴美珍. 大黄附子汤中诸药的不同组合及煎法对乌头碱含量的影响. 中国药房 14, 634-635 (2003). | |
|  | 2 | 路杰, 高姗姗, 张晓丹,闫静. 八味地黄方与人参汤共煎液毒性的实验研究. 中医药信息 28, 24-27 (2011). | |
|  | 3 | 钟梅芳. 麻黄附子甘草汤的不同配伍方式对其毒性成分的影响分析. 实用中医内科杂志 26, 68-69 (2012). | |
|  | 4 | 肖勇, 马增春, 王宇光, 谭洪玲, 赵永红, 梁乾德, 汤响林, 肖成荣,高月. 参附注射液配伍对乌头碱诱发心律失常的减毒研究. 中药药理与临床 29, 12-15 (2013). | |
|  | 5 | 王传娟. 麻黄附子甘草汤同时煎煮的减毒效果观察. 世界最新医学信息文摘（连续型电子期刊）, 32-33 (2015). | |
|  | 6 | 熊永爱, 黄勤挽,杨明. 四逆汤及其拆方心脏毒性研究. 中医杂志 54, 2035-2038 (2013). | |
|  | 7 | 杨海润, 孙建宁, 张广平, 朱晓光, 张思玉,解素花. 四逆汤组方不同配伍毒效关系研究. 中国实验方剂学杂志 19, 266-269 (2013). | |
|  | 8 | 张广平, 朱晓光, 孟贺, 张思玉, 杨海润,叶祖光. 四逆汤组方配伍减毒实验研究. 中国中医药信息杂志, 29-31 (2013). | |
|  | 9 | 吴康衡. 关白附、松节、苦檀子中毒解救方. 东方药膳 (2013). | |
|  | 10 | 潘宗奇,黄遂和. 降金温水治疗鼻咽癌放射性口腔炎1例. in 世界中医药学会联合会肿瘤经方治疗研究专业委员会成立大会暨第一届学术年会论文集 327-330 (北京, 2015). | |
|  | 11 | 余利华. 经方“热因热用”治疗发热验案两则. 成都中医药大学学报 36, 110-111 (2013). | |
|  | 12 | 汪戎锦, 丁茹,刘忠英. 基于三重四级杆串联质谱技术研究甘草附子汤中乌头类生物碱的吸收转运机制. in 第三届全国质谱分析学术报告会论文集 316-316 (厦门, 2017). | |
|  | 13 | 王鹏一雄. 浅析桂枝附子汤煎煮时间对改善心肌缺血功效的影响. 名医 000, P.140-140 (2018). | |
|  | 14 | 王彩云,万龙. 麻黄附子甘草汤的不同配伍方式对其毒性成分的影响. 中国医药指南 14, 37-38 (2016). | |
|  | 15 | 尹玉平. 补阳还五汤+针刺联合西药治疗脑梗塞随机平行对照研究. 实用中医内科杂志 31, 24-26 (2017). | |
|  | 16 | 肖建平, 朱来来,朱毛. 车苓附子汤治疗慢性肺源性心脏病心衰阳虚水泛证48例. 浙江中医杂志 55, 252-253 (2020). | |
|  | 17 | 李莹, et al. 四逆汤中甘草减附子之毒的代谢组学研究. 中国中药杂志 41, 1523-1529 (2016). | |
|  | 18 | 程丽飞, 王华林 & 李敏. 四逆汤及其拆方水煎液对缺血心肌细胞的保护作用. 西部中医药 32, 9-12 (2019). | |
|  | 19 | 鞠爱霞, 张曦文, 孙爽, 张羽 & 李秋红. 基于CYP450酶的四逆汤复方配伍减毒机制研究. 时珍国医国药 29, 1863-1866 (2018). | |
|  | 20 | 李志勇, et al. 四逆汤配伍环境下的附子“效-毒网络”交集调控研究. 中国中药杂志 40, 733-738 (2015). | |
|  | 21 | 贾玉. 大黄附子汤中附子生用与制用的药效及毒性. 医药与保健, 105-106 (2015). | |
|  | 22 | 胡丽萍, 张惠颖 & 马杰. 温经颗粒动物长期毒性试验. 毒理学杂志 21(2007). | |
|  | 23 | 乔艳. 麻黄附子甘草汤同时煎煮的减毒效果观察. 北方药学 17, 91-92 (2020). | |
|  | 24 | 裴妙荣, 王世民,李晶. 四逆汤中甘草对附子解毒作用的相关性分析. 中国中药杂志, 50-52,65 (1996). | |
| Fuzilizhong Pills | 1 | 王建凯,刘国良. 附子理中丸致中毒反应2例报道. 实用中医药杂志 17, 43 (2001). | |
|  | 2 | 张向力. 附子理中丸中毒致心律失常1例. 中国中医药信息杂志 3(1996). | |
|  | 3 | 齐敏. 服附子理中丸致中毒1例报告. 内蒙古中医药 0(1994). | |
|  | 4 | 张庆辉. 附子理中丸中毒致心律失常1例. 临床荟萃 25, 2082 (2010). | |
|  | 5 | 杨春燕, 薛志红,姚凯. 附子理中汤超微颗粒与传统饮片的毒性对比研究. 赤峰学院学报（自然科学版） 33, 163-165 (2017). | |
|  | 6 | 杨晓颖. 附子理中丸临床用药情况调查分析. 临床合理用药杂志 11, 98-100 (2018). | |
|  | 7 | 黄献钟. 扶阳健脾法减轻结肠癌化疗毒副反应临床研究. 亚太传统医药 12, 131-132 (2016). | |
|  | 8 | 黄仕沛. 持续三月不明原因高热不退,"一剂知"案. in 广州2014年中医药学术年会论文集 224-226 (广州, 2014). | |
|  | 9 | 刘洋, 董世芬, 张硕峰,孙建宁. 配伍对附子理中丸毒性及药效影响的实验研究. in 2012年全国有毒中药的研究及其合理应用交流研讨会论文集 | |
|  | 10 | 陈玉秋. 附子理中丸中毒致严重休克心律失常l例. 吉林医学信息 019, 42 (2002). | |
|  | 11 | 万莉华. 附子理中汤对老年抗生素相关性腹泻疗效的临床研究. 中国医疗设备 32(2017). | |
|  | 12 | 何熙果,张臻,尹恒.附子理中汤“减毒增效”现代研究概况[J].中药与临床,2017,8(01):62-65. | |
| The wine soaked with aconite | 1 | 吴秋枫. 附子酒致心律失常1例. 浙江中西医结合杂志 12(2002). | |
|  | 2 | 凌巍山,孟庆国.抢救乌头碱中毒五例[J].佳木斯医学院学报,1994(04):87. | |
|  | 3 | 楼亚梅.重度乌头碱中毒致室性快速心律失常的急救护理[J].护理与康复,2004(02):142-143. | |
|  | 4 | 钟希文,梅全喜,张文霞.试论《肘后备急方》中附子的应用[J].时珍国医国药,2016,27(01):171-173. | |
|  | 5 | 高悦,李飞. 近10年附子临床中毒病例分析[A]. 中华中医药学会.2010中药炮制技术、学术交流暨产业发展高峰论坛论文集[C].中华中医药学会:中华中医药学会,2010:5. | |
| Salted aconite | 1 | 熊秋韵,张慧琼,谢晓芳,刘怡,熊亮,邓青秀,彭成.附子不同炮制品对风湿寒痹类风湿关节炎大鼠的急性毒性试验研究[J].中药药理与临床,2020,36(02):149-158. | |
|  | 2 | 郑文彩.中药炮制过程中减毒方法的临床分析[J].中外医疗,2019,38(22):163-165. | |
|  | 3 | 唐欣,谢晓芳,王文林,饶朝龙,彭成.附子对妊娠虚寒腹痛模型大鼠的毒效研究[J].中药药理与临床,2019,35(03):101-105. | |
|  | 4 | 刘红梅. 胆巴炮制对附子化学成分及神经毒性影响[D].成都中医药大学,2018. | |
|  | 5 | 国伟,谭鹏,费淑琳,秦语欣,陈炯,吴月娇,赵玲玲,李飞.炮制对淡附子中3种双酯型生物碱及其水解产物的影响[J].河南中医,2016,36(06):1096-1099. | |
|  | 6 | 国伟,谭鹏,吴月娇,秦语欣,赵玲玲,李飞.双辅料炮制对盐附子传统性状和酯型生物碱的影响[J].中成药,2015,37(06):1289-1293. | |
|  | 7 | 柴玉爽,王玉刚,花雷,张聿梅,肖新月,詹宏磊,雷帆,万红娇,林瑞超,邢东明,杜力军.附子乌头草乌及其炮制品的毒效比较[J].世界科学技术(中医药现代化),2011,13(05):847-851. | |
|  | 8 | 陈红钰,王亚其,潘黎,卢豪,严光焰,李宏霞.盐附子遗传毒性研究[J].现代预防医学,2009,36(05):917-919. | |
|  | 9 | 陈红钰,王亚其,潘黎,卢豪,李宏霞.盐附子的遗传毒性研究[J].毒理学杂志,2007(04):340. | |
|  | 10 | 王冲,严光焰,何晓娟,王莉,李宏霞.盐附子对小鼠的急性神经毒性作用[J].华西药学杂志,2007(03):300-301. | |
|  | 11 | 王冲, 严光焰, 何晓娟, 王莉,李宏霞. 盐附子对小鼠的急性神经毒性作用. 华西药学杂志 22, 300-301 (2007). | |
| Prepared aconite | 1 | 肖凯. 乌头类中药大鼠胚胎发育毒性体内外试验研究[D].四川大学,2006. | |
|  | 2 | 沈桢巍, 冯兰英,徐红. 乌头碱中毒致严重心律失常二例. 中华心律失常学杂志 5, 370-370 (2001). | |
|  | 3 | 吴玲毓. 附片中毒致心律失常3例报告. 中国社区医师(综合版), 68 (2006). | |
|  | 4 | 马跃峰, 郭成林, 马永林, 覃建林, 杜晓莉, 陆荣生, 陆仟,黄旭光. 氯嘧磺隆与2甲4氯钠盐混配对香附子的联合毒力测定. in 第十二届全国杂草科学大会 8 (中国山西太原, 2015). | |
|  | 5 | 马斌智. 超常规剂量苍耳子中毒致室速1例报告. 青海医药杂志 (2013). | |
|  | 6 |  | |
|  | 7 | 张爱萍. 制附片中毒致心律失常及四相完全性右束支传导阻滞1例. 现代中西医结合杂志 12(2003). | |
|  | 8 | 谭勇. 博士, 中国中医科学院 (2009). | |
|  | 9 | 熊静悦, 牟道华, 唐大轩,谭正怀. 白附子急性毒性作用研究. 四川生理科学杂志 32, 101-103 (2010). | |
|  | 10 | 王金凤,蔡兴东. 白附片中毒三例报告. 北方药学 08, 128-128 (2011). | |
|  | 11 | 文永兰, 李亚玲,李俊. 白附片中毒一例. 实用药物与临床 12, 435 (2009). | |
|  | 12 | 杨永莲. 附片引起不良反应2例. 中外医学研究, 156 (2013). | |
|  | 13 | 罗仁书,何治勇. 白附片中毒致心动过速及房室早搏1例. 实用中医药杂志 32, 274 (2016). | |
|  | 14 | 钱晚. 煎煮时间不够 中药变“毒药”. 恋爱婚姻家庭(养生), 33 (2016). | |
|  | 15 | 王新月. 附子蓄积中毒例析. 中医函授通讯 16(1997). | |
|  | 16 | 张万英. 乌头碱致急性中毒一例报告. 青海医药杂志 37, 49 (2007). | |
|  | 17 | 吴玲毓. 附片中毒致心律失常3例报告. 中国社区医师(综合版), 68 (2006). | |
|  | 18 | 张风雷, 李立纪, 吴荣祖,刘培儒. 附子、附片及煎煮液化学成分比较实验及毒性研究. 云南中医中药杂志 25, 29-31 (2004). | |
|  | 19 | 段小花, et al. 不同剂量和煎煮时间的附子煎剂对小鼠非特异性免疫和毒性的影响. 时珍国医国药 30, 345-347 (2019). | |
|  | 20 | 黄梦婷, et al. 服用附片中毒引起Ⅲ度房室传导阻滞及凝血功能异常1例报道. 现代医药卫生 33, 3210-3211 (2017). | |
|  | 21 | 女子喝附片羊肉汤后中毒 附片本身有毒需煮一小时以上. 中国食品, 150 (2016). | |
|  | 22 | 陈泣, 韩飞 & 龚千锋. 基于樟帮特色炮制品-附子临江片补阳药效研究. 时珍国医国药 27, 2644-2646 (2016). | |
|  | 23 | 陈丹丹, et al. 广东省中医院含附子饮片的处方分析. 中药材 42, 449-452 (2019). | |
|  | 24 | 李纯青. 大剂量附片、干姜加重大疱性类天疱疮1例. 皮肤病与性病 24(2002). | |
|  | 25 | 韩勇. 附片中毒致心室纤颤抢救成功1例. 现代医药卫生 22(2006). | |
|  | 26 | 张秀, 黄伟, 赵科, 向菊芳, 刘琪, 曹方引, 周雪,谭正怀. 不同炮制工艺对附片毒性及功效的影响. 中药药理与临床 35, 103-107 (2019). | |
|  | 27 | 朱小珊, 刘刚, 肖二,梅之南. 傣药大黑附子的毒理学研究. in 2009年传统医药国际科技大会论文集 361-365 (广州, 2009). | |
|  | 28 | 蔡徐骄. 硕士, 成都中医药大学 (2007). | |
|  | 29 | 张媛. 硕士, 成都中医药大学 (2008). | |
|  | 30 | 余成浩. 博士, 成都中医药大学 (2006). | |
|  | 31 | 周静波. 博士, 成都中医药大学 (2009). | |
|  | 32 | 余葱葱. 博士, 成都中医药大学 (2009). | |
|  | 33 | 周思思. 硕士, 安徽医科大学 (2013). | |
|  | 34 | 肖勇. 硕士, 中南大学 (2014). | |
|  | 35 | 沈勇. 硕士, 福州大学 (2014). | |
|  | 36 | 彭稳稳. 硕士, 南京中医药大学 (2013). | |
|  | 37 | 尤青. 硕士, 中南大学 (2014). | |
|  | 38 | 李莹. 博士, 成都中医药大学 (2013). | |
|  | 39 | 陈秋薇. 硕士, 成都中医药大学 (2014). | |
|  | 40 | 李静. 硕士, 山西中医学院 (2015). | |
|  | 41 | 邱丽丽. 博士, 山东中医药大学 (2019). | |
|  | 42 | 谢运飞. 博士, 成都中医药大学 (2019). | |
|  | 43 | 余婷. 硕士, 湖北中医药大学 (2019). | |
|  | 44 | 宋帅. 博士, 南方医科大学 (2016). | |
|  | 45 | 裴妙荣,赵丽娜. 大黄附子汤中附子生用与制用的药效及毒性研究. 中药药理与临床 24, 4-5 (2008). | |

**Table S3 The number of literatures of decoction time in the DLTA.**

| DECOCTION TIME | NUM | ARTICLES |
| --- | --- | --- |
| <30min（7） | 1 | 罗显田. 附子中毒致恶性心律失常、心脏骤停1例报道. 海军医学杂志 (2000). |
|  | 2 | 曹昌雪.附子中毒1例报道. 实用中医药杂志 28, 705 (2012). |
|  | 3 | 李蕙君.附子中毒致严重心律失常2例. 中国实用内科杂志 23(2003). |
|  | 4 | 陈建宗, 黄晨, 高建苑, 田季雨,奚淑芳. 中药附子中毒引起心律失常10例. 药物流行病学杂志 13, 223-224 (2004). |
|  | 5 | 文永兰, 李亚玲,李俊. 白附片中毒一例. 实用药物与临床 12, 435 (2009). |
|  | 6 | 时英菊, 石晓华,陈会娟. 生附子中毒急救1例护理体会. 河北中医 33, 1731 (2011). |
|  | 7 | 侯胜福, 张湘兰,林春. 中药附子急性中毒1例. 临床合理用药杂志 04, 73-73 (2011). |
| 1h-2h（15） | 1 | 吴玲毓. 附片中毒致心律失常3例报告. 中国社区医师(综合版), 68 (2006). |
|  | 2 | 王宁波,张学臻. 附子中毒致严重心律失常1例. 医学文选 18, 1006 (1999). |
|  | 3 | 王义臣,王尽美. 口服中药附子煎剂致严重中毒1例. 中国医学创新 6, 50 (2009). |
|  | 4 | 时英菊, 石晓华,陈会娟. 生附子中毒急救1例护理体会. 河北中医 33, 1731 (2011). |
|  | 5 | 秦婉玲,施恒. 炙甘草汤治疗附子中毒一例. in 江西省中西医结合学会第九次活血化瘀学术研讨会活血化瘀临床应用新进展培训班论文集 (中国江西南昌, 2011). |
|  | 6 | 廖显春.乌头碱中毒致非阵发性室性心动过速1例报告. 岭南急诊医学杂志 6, 232-233 (2001). |
|  | 7 | 陈建宗,黄晨, 高建苑, 田季雨,奚淑芳. 中药附子中毒引起心律失常10例. 药物流行病学杂志 13, |
|  | 8 | 游书烟. 附子中毒反应1例. 天津中医药大学学报 25(2006). |
|  | 9 | 许廷生. 附子临床应用不良反应的分析及对策. 中国社区医师：医学专业 5(2003). |
|  | 10 | 李蕙君. 附子中毒致严重心律失常2例. 中国实用内科杂志 23(2003). |
|  | 11 | 余葱葱, 郭力 ,彭成. 不同煎煮时间的附子毒效组分与毒性成分质量控制研究. in 中华中医药学会2008临床中药学学术研讨会论文集 229-234 (北京, 2008). |
|  | 12 | 钱晚. 煎煮时间不够 中药变“毒药”. 恋爱婚姻家庭(养生), 33 (2016). |
|  | 13 | 徐晓艳,汪永锋.李可弟子吕英主任临床使用含附子汤剂的煎煮方法浅析[J].甘肃中医学院学报,2012,29(06):72-73. |
|  | 14 | 贾雪岩. 附子临床应用安全评价研究[D].广州中医药大学,2016. |
|  | 15 | 刘红梅. 胆巴炮制对附子化学成分及神经毒性影响[D].成都中医药大学,2018. |
| 2-2.5h | 1 | 尚翠香, 乔丽红, 王晓红,袁欢. 综合措施救治附子中毒致心跳呼吸骤停1例. 陕西中医 30, 1396 (2009). |
|  | 2 | 叶俊玲,晏子友,王茂泓,李罗德.附子救治急危重症临床运用探析[J].四川中医,2012,30(01):52-54. |
|  | 3 | 罗昌国,张瑞贤.对附子大剂量应用毒性问题的探讨[J].中国中药杂志,2009,34(18):2423-2424. |
|  | 4 | 黄钰玲.中西医结合救治白附子中毒1例报告. 新中医 39, 78-78 (2007). |
| 3h | 1 | 褟湘珍,芦承有. 附子中毒所致阿-斯二氏综合征一例报告. 中华内科杂志 13, 1001 (1965). |
|  | 2 | 傅文录.吴佩衡应用附子的经验[J].河南中医,2011,31(04):339-341. |
| >3h | 1 | 张万英. 乌头碱致急性中毒一例报告. 青海医药杂志 37, 49 (2007). |
|  | 2 | 胡春勤,周水金,王昆.附子煎煮时间、给药剂量与温阳功效的关系[J].当代医学,2017,23(19):69-70. |
|  | 3 | 谭婉莹,夏勇,李艳苹,皮强中,罗素新.附子心脏毒-效物质基础及相关作用机理研究[J].中药药理与临床,2019,35(03):95-101. |
|  | 4 | 徐勤芳,顾文忠.超大剂量附子、苍术治疗重症阳虚寒湿型强直性脊柱炎验案1则[J].上海中医药杂志,2016,50(09):33-35. |

**Table S4 The number of literatures on different dosages of aconite in DLTA.**

| dosage | NUM | articles |
| --- | --- | --- |
| 1-15g/d（10） | 1 | 陈理书, 张云鹏,杨悦娅. 临床应用附子78例探析. 辽宁中医杂志 32, 348-349 (2005). |
|  | 2 | 郭效建, 刘安述,楮体云. 半夏、附子配伍中毒2例报告. 山东医药 44, 75 (2004). |
|  | 3 | 陈建宗, 黄晨, 高建苑, 田季雨,奚淑芳. 中药附子中毒引起心律失常10例. 药物流行病学杂志 13, 223-224 (2004). |
|  | 4 | 张爱萍. 制附片中毒致心律失常及四相完全性右束支传导阻滞1例. 现代中西医结合杂志 12(2003). |
|  | 5 | 游敏生. 附子中毒1例报告. 中华临床医药杂志（北京） 4(2003). |
|  | 6 | 许廷生. 附子临床应用不良反应的分析及对策. 中国社区医师：医学专业 5(2003). |
|  | 7 | 沈桢巍, 冯兰英,徐红. 乌头碱中毒致严重心律失常二例. 中华心律失常学杂志 5, 370-370 (2001). |
|  | 8 | 罗显田. 附子中毒致恶性心律失常、心脏骤停1例报道. 海军医学杂志 (2000). |
|  | 9 | 何忠文. 附子,川乌,草乌中毒12例辨析. 江西中医学院学报 12(2000). |
|  | 10 | 刘翠华,初振仁. 半夏与附子配伍应用的临床观察. 吉林中医药, 35 (1989). |
| 16-30g/d（8） | 1 | 吴玲毓. 附片中毒致心律失常3例报告. 中国社区医师(综合版), 68 (2006). |
|  | 2 | 游敏生. 附子中毒1例报告. 中华临床医药杂志（北京） 4(2003). |
|  | 3 | 彭澍. 急性附子中毒1例. 中国实用乡村医生杂志 17, 57-58 (2010). |
|  | 4 | 王义臣,王尽美. 口服中药附子煎剂致严重中毒1例. 中国医学创新 6, 50 (2009). |
|  | 5 | 侯胜福, 张湘兰 & 林春. 中药附子急性中毒1例. 临床合理用药杂志 04, 73-73 (2011). |
|  | 6 | 梁小坤. 急性附子中毒23例临床分析. 临床医药实践B版 19, 745-746 (2010). |
|  | 7 | 曹昌雪. 附子中毒1例报道. 实用中医药杂志 28, 705 (2012). |
|  | 8 | 廖显春. 乌头碱中毒致非阵发性室性心动过速1例报告. 岭南急诊医学杂志 6, 232-233 (2001). |
| 31-50g/d（16） | 1 | 吴玲毓. 附片中毒致心律失常3例报告. 中国社区医师(综合版), 68 (2006). |
|  | 2 | 王宁波,张学臻. 附子中毒致严重心律失常1例. 医学文选 18, 1006 (1999). |
|  | 3 | 文永兰, 李亚玲,李俊. 白附片中毒一例. 实用药物与临床 12, 435 (2009). |
|  | 4 | 时英菊, 石晓华,陈会娟. 生附子中毒急救1例护理体会. 河北中医 33, 1731 (2011). |
|  | 5 | 乐永宏. 急性附子中毒伴胆碱酯酶活性抑制1例. 中国误诊学杂志 1, 154 (2001). |
|  | 6 | 黄钰玲. 中西医结合救治白附子中毒1例报告. 新中医 39, 78-78 (2007). |
|  | 7 | 何玉华. 乌头中毒1例的救治. 药物不良反应杂志 9, 193-193 (2007). |
|  | 8 | 何忠文. 附子,川乌,草乌中毒12例辨析. 江西中医学院学报 12(2000). |
|  | 9 | 陈添连, 廖金龙,钟招兰. 1例超量服附片中毒患者的抢救与护理体会. 临床医学工程 17, 111-112 (2010). |
|  | 10 | 徐勤芳,顾文忠.超大剂量附子、苍术治疗重症阳虚寒湿型强直性脊柱炎验案1则[J].上海中医药杂志,2016,50(09):33-35. |
|  | 11 | 陈嘉斌,陈伟,徐国暑,王胜,胡琴琴,施云福,余志红,柴可群.大剂量附子治疗晚期恶性肿瘤的安全性评价及用药规律分析[J].中医肿瘤学杂志,2020,2(04):23-28. |
|  | 12 | 郝巧英,孙银红.超剂量使用中药饮片原因分析[J].世界最新医学信息文摘,2019,19(22):187+189. |
|  | 13 | 杨雪,夏东胜,田春华,李岚,孙毅.508例附子不良反应文献分析[J].中国药物警戒,2017,14(10):615-621. |
|  | 14 | 薛兆坤,沈海滨.大剂量附子临床使用安全性分析[J].内蒙古中医药,2017,36(16):96-97. |
|  | 15 | 刘华平,李兆荣,吴涛,史锁芳.浅谈超剂量使用中药饮片[J].中医药导报,2015,21(16):50-51. |
|  | 16 | 张金莲,曾昭君,张冰,余书琦.附子临床不良反应分析[J].中国实验方剂学杂志,2014,20(18):228-231. |
| more than 50g/d（11） | 1 | 王金凤,蔡兴东. 白附片中毒三例报告. 北方药学 08, 128-128 (2011). |
|  | 2 | 尚翠香, 乔丽红, 王晓红,袁欢. 综合措施救治附子中毒致心跳呼吸骤停1例. 陕西中医 30, 1396 (2009). |
|  | 3 | 高雪英,张丽芬. 附子中毒报告1例. 光明中医 25, 2109-2110 (2010). |
|  | 4 | 黄兆玉. 中药附子中毒一例报告. 青海医药杂志 37, 13 (2007). |
|  | 5 | 游书烟. 附子中毒反应1例. 天津中医药大学学报 25(2006). |
|  | 6 | 吴玲毓. 附片中毒致心律失常3例报告. 中国社区医师(综合版), 68 (2006). |
|  | 7 | 许廷生. 附子临床应用不良反应的分析及对策. 中国社区医师：医学专业 5(2003). |
|  | 8 | 李纯青. 大剂量附片、干姜加重大疱性类天疱疮1例. 皮肤病与性病 24(2002). |
|  | 9 | 白锦栋. 长期大剂量使用附子一例. 神经药理学报 (1989). |
|  | 10 | 刘玉庆,戴雁彦. 附子中毒1例. in 第二届长城国际中西医结合心脏病论坛论文集 168-170 (北京, 2011). |
|  | 11 | 梁文坚, 王清海, 李典鸿,杨跃娜. 附子中毒致恶性心律失常1例报道. 按摩与康复医学, 119-119,120 (2014). |

**Table S5 The number of reports on the causes of aconite poisoning in DLTA**

| Other influencing factors | NUMBER | articles |
| --- | --- | --- |
| drug-induced side effects or unspecified | 1 | 王仁轼. 常规剂量附片中毒致恶性心律失常1例. 四川医学 22, 509 (2001). |
|  | 2 | 黄梦婷, et al. 服用附片中毒引起Ⅲ度房室传导阻滞及凝血功能异常1例报道. 现代医药卫生 33, 3210-3211 (2017). |
|  | 3 | 蔡全莒. 乌头类中药致不良反应一例报告. 青海医药杂志 46, 64 (2016). |
|  | 4 | 李成皇. 乌头碱中毒致心律失常1例报告. 中国保健营养（下旬刊） 23, 1166-1166 (2013). |
|  | 5 | 李永哲,秦孝智. 附子中毒致严重心律失常1例. 医学理论与实践 23, 760 (2010). |
|  | 6 | 曹昌雪. 附子中毒1例报道. 实用中医药杂志 28, 705 (2012). |
|  | 7 | 乐永宏. 急性附子中毒伴胆碱酯酶活性抑制1例. 中国误诊学杂志 1, 154 (2001). |
|  | 8 | 陈玉秋. 附子理中丸中毒致严重休克心律失常l例. 吉林医学信息 019, 42 (2002). |
|  | 9 | 李蕙君. 附子中毒致严重心律失常2例. 中国实用内科杂志 23(2003). |
|  | 10 | 郭效建, 刘安述,楮体云. 半夏、附子配伍中毒2例报告. 山东医药 44, 75 (2004). |
|  | 11 | 何少峰. 小剂量附子中毒致恶性心律失常、心搏骤停1例. 中国煤炭工业医学杂志 8(2005). |
|  | 12 | 余葱葱, 郭力,彭成. 不同煎煮时间的附子毒效组分与毒性成分质量控制研究. in 中华中医药学会2008临床中药学学术研讨会论文集 229-234 (北京, 2008). |
|  | 13 | 张向力. 附子理中丸中毒致心律失常1例. 中国中医药信息杂志 3(1996). |
|  | 14 | 王雅娜. 中西医结合抢救乌头类中药中毒5例. 中国中西医结合杂志 0(1994). |
|  | 15 | 齐敏. 服附子理中丸致中毒1例报告. 内蒙古中医药 0(1994). |
|  | 16 | 周长绵,黄健. 抢救急性附子中毒8例临床报告. 新中医, 39-40 (1991). |
|  | 17 | 党中文. 65例乌头碱中毒临床分析. 云南医药 33, 410-411 (2012). |
|  | 18 | 王金凤,蔡兴东. 白附片中毒三例报告. 北方药学 08, 128-128 (2011). |
|  | 19 | 庄会江. 附子急性中毒16例救治体会. 中国实用医药 4, 185-186 (2009). |
|  | 20 | 蔡吉芬, 杨云贵, 周丽琼, 罗亚坤,董家能. 附子中毒156例临床分析. 昆明医学院学报 32, 131-132 (2011). |
|  | 21 | 吴鹏起. 急性附子中毒20例救治体会. 吉林医学 33, 1942-1943 (2012). |
|  | 22 | 梁小坤. 急性附子中毒23例临床分析. 临床医药实践B版 19, 745-746 (2010). |
|  | 23 | 侯胜福, 张湘兰,林春. 中药附子急性中毒1例. 临床合理用药杂志 04, 73-73 (2011). |
|  | 24 | 杨永莲. 附片引起不良反应2例. 中外医学研究, 156 (2013). |
|  | 25 | 侯静. 急救1例附子中毒病人后的护理体会. in 江西省第八次中西医结合危重病、急救医学学术研讨会论文集 (中国江西赣州, 2013). |
|  | 26 | 久服附子中毒案. 上海中医药杂志 48, 32 (2014). |
|  | 27 | 蔡全莒. 乌头类中药致不良反应一例报告. 青海医药杂志 46, 64 (2016). |
|  | 28 | 韩丹阳, 李蕊白,赵勇. 参附注射液静脉滴注致双下肢疼痛伴震颤一例. 中西医结合心血管病电子杂志 4, 115,117 (2016). |
|  | 29 | 彭文鹏. 附子所致中毒性心肌炎一例. 饮食保健 5, 82-83 (2018). |
|  | 30 | 张丽红, 施青青,孙婧. 乌头碱中毒疑似急性冠脉综合征1例. 武警医学 30, 992-993 (2019). |
|  | 31 | 刘玉玲. 附子(乌头碱)中毒治愈成功1例. 特别健康, 63 (2020). |
|  | 32 | 钱晚. 煎煮时间不够 中药变“毒药”. 恋爱婚姻家庭(养生), 33 (2016). |
|  | 33 | 杨翠荣. 乌头类中药37例临床中毒反应及治疗分析. 实用中西医结合临床 17, 161-162 (2017). |
|  | 34 | 李朝容,彭文静. 服用常规剂量附片中毒致二度房室传导阻滞1例. 基层医学论坛 24, 1467-1468 (2020). |
|  | 35 | 贾雪岩. 附子临床应用安全评价研究[D].广州中医药大学,2016. |
|  | 36 | 马菲. 附子复方治疗类风湿关节炎寒湿阻络证的有效性和安全性的临床研究[D].北京中医药大学,2013. |
|  | 37 | 田玉静. 附子临床应用安全性的前瞻性研究[D].广州中医药大学,2010. |
|  | 38 | 刘玉,唐雪春.附子应用安全性的研究进展[J].辽宁中医药大学学报,2009,11(05):56-58. |
| improper processing | 1 | 韩勇. 附片中毒致心室纤颤抢救成功1例. 现代医药卫生 22(2006). |
|  | 2 | 廖显春. 乌头碱中毒致非阵发性室性心动过速1例报告. 岭南急诊医学杂志 6, 232-233 (2001). |
|  | 3 | 侯胜福, 张湘兰,林春. 中药附子急性中毒1例. 临床合理用药杂志 04, 73-73 (2011). |
|  | 4 | 蒋志青. 乌头、附子引起急性中毒反应1例临床探讨. 黑龙江中医药 43, 24 (2014). |
|  | 5 | 丘小惠,何洁. 煎煮时间及甘草配伍剂量对附子中酯型生物碱含量的影响. 时珍国医国药 18, 3015-3017 (2007). |
|  | 6 | 王良馥,陈自力. 综合抢救重度附子中毒7例. 中国中医药信息杂志 12, 86-87 (2005). |
|  | 7 | 宋友湘. 附子中毒致恶性心律失常分析. 国际医药卫生导报 (2005). |
|  | 8 | 张爱萍. 制附片中毒致心律失常及四相完全性右束支传导阻滞1例. 现代中西医结合杂志 12(2003). |
|  | 9 | 张向力. 附子理中丸中毒致心律失常1例. 中国中医药信息杂志 3(1996). |
|  | 10 | 蒋志青. 乌头、附子引起急性中毒反应1例临床探讨. 黑龙江中医药 43, 24 (2014). |
| accumulation | 1 | 王新月. 附子蓄积中毒例析. 中医函授通讯 16(1997). |
|  | 2 | 李纯青. 大剂量附片、干姜加重大疱性类天疱疮1例. 皮肤病与性病 24(2002). |
|  | 3 | 白锦栋. 长期大剂量使用附子一例. 神经药理学报 (1989). |
|  | 4 | 高雪英 ,张丽芬. 附子中毒报告1例. 光明中医 25, 2109-2110 (2010). |
|  | 5 | 梁文坚, 王清海, 李典鸿 ,杨跃娜. 附子中毒致恶性心律失常1例报道. 按摩与康复医学, |
|  | 6 | 久服附子中毒案. 上海中医药杂志 48, 32 (2014). |
|  | 7 | 陈金月, 周芳,黄世优. 大剂量使用附子的安全性研究. 亚太传统医药 4, 37-39 (2008). |
|  | 8 | 久服附子中毒案. 上海中医药杂志 48, 32 (2014). |
| aconite medicated with wine/food | 1 | 韩勇. 附片中毒致心室纤颤抢救成功1例. 现代医药卫生 22(2006). |
|  | 2 | 王良馥,陈自力. 综合抢救重度附子中毒7例. 中国中医药信息杂志 12, 86-87 (2005). |
|  | 3 | 许廷生. 附子临床应用不良反应的分析及对策. 中国社区医师：医学专业 5(2003). |
|  | 4 | 李蕙君. 附子中毒致严重心律失常2例. 中国实用内科杂志 23(2003). |
|  | 5 | 吴秋枫. 附子酒致心律失常1例. 浙江中西医结合杂志 12(2002). |
|  | 6 | 唐春荣. 附子中毒1例. 中国中药杂志 27(2002). |
| Eating disorders/autonomous diseases | 1 | 丘小惠,何洁. 煎煮时间及甘草配伍剂量对附子中酯型生物碱含量的影响. 时珍国医国药 18, 3015-3017 (2007). |
|  | 2 | 黄兆玉. 中药附子中毒一例报告. 青海医药杂志 37, 13 (2007). |
|  | 3 | 郭效建, 刘安述,楮体云. 半夏、附子配伍中毒2例报告. 山东医药 44, 75 (2004). |
|  | 4 | 曹昌雪. 附子中毒1例报道. 实用中医药杂志 28, 705 (2012). |
|  | 5 | 时英菊,石晓华,陈会娟.生附子中毒急救1例护理体会[J].河北中医,2011,33(11):1731. |
|  | 6 | 高雪英,张丽芬. 附子中毒报告1例. 光明中医 25, 2109-2110 (2010). |
